# Engineering Fe-N_4_ Electronic Structure with Adjacent Co-N_2_C_2_ and Co Nanoclusters on Carbon Nanotubes for Efficient Oxygen Electrocatalysis

**DOI:** 10.1007/s40820-023-01195-2

**Published:** 2023-10-20

**Authors:** Mingjie Wu, Xiaohua Yang, Xun Cui, Ning Chen, Lei Du, Mohamed Cherif, Fu-Kuo Chiang, Yuren Wen, Amir Hassanpour, François Vidal, Sasha Omanovic, Yingkui Yang, Shuhui Sun, Gaixia Zhang

**Affiliations:** 1 State Key Laboratory of New Textile Materials and Advanced Processing Technologies, Wuhan Textile University, Wuhan, 430200 People’s Republic of China; 2 Institut National de la Recherche Scientifique (INRS), Center Énergie Matériaux Télécommunications, Varennes, QC J3X 1P7 Canada; 3 Department of Chemical Engineering, McGill University, 3610 University Street, Montreal, QC H3A 0C5 Canada; 4 Department of Electrical Engineering, École de Technologie Supérieure (ÉTS), Montreal, QC H3C 1K3 Canada; 5 Canadian Light Source (CLS), 44 Innovation Boulevard, Saskatoon, SK S7N 2V3 Canada; 6 National Institute of Low-Carbon-and-Clean-Energy, Beijing, 102211 People’s Republic of China; 7 School of Materials Science and Engineering, University of Science and Technology Beijing, Beijing, 100083 People’s Republic of China

**Keywords:** Atomically dispersed, Fe–N–C catalysts, ORR/OER, Rechargeable zinc-air battery, Fuel cells

## Abstract

Regulating the local configuration of atomically dispersed transition-metal atom catalysts is the key to oxygen electrocatalysis performance enhancement. Unlike the previously reported single-atom or dual-atom configurations, we designed a new type of binary-atom catalyst, through engineering Fe-N_4_ electronic structure with adjacent Co-N_2_C_2_ and nitrogen-coordinated Co nanoclusters, as oxygen electrocatalysts. The resultant optimized electronic structure of the Fe-N_4_ active center favors the binding capability of intermediates and enhances oxygen reduction reaction (ORR) activity in both alkaline and acid conditions. In addition, anchoring M–N–C atomic sites on highly graphitized carbon supports guarantees of efficient charge- and mass-transports, and escorts the high bifunctional catalytic activity of the entire catalyst. Further, through the combination of electrochemical studies and in-situ X-ray absorption spectroscopy analyses, the ORR degradation mechanisms under highly oxidative conditions during oxygen evolution reaction processes were revealed. This work developed a new binary-atom catalyst and systematically investigates the effect of highly oxidative environments on ORR electrochemical behavior. It demonstrates the strategy for facilitating oxygen electrocatalytic activity and stability of the atomically dispersed M–N–C catalysts.

**Supplementary Information:**

The online version contains supplementary material available at 10.1007/s40820-023-01195-2.

## Highlights

- Engineering Fe-N_4_ electronic structure with adjacent Co-N_2_C_2_ and Co sub-nanoclusters to optimize the electronic structure of the Fe-N_4_ active center is first demonstrated.
- Electrochemical studies reveal that the amorphous carbon corrosion leads to significantly increased charge- and mass-transport resistances.
- The operando X-ray absorption spectroscopy allows an investigation of the effect of highly oxidative environments on electrochemical behavior.

**Supplementary Information:**

The online version contains supplementary material available at 10.1007/s40820-023-01195-2.

## Graphical abstract

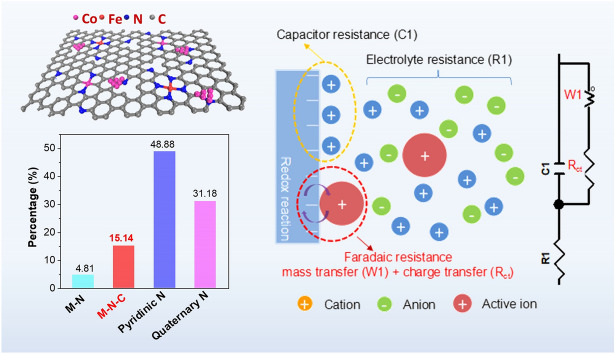

## Introduction

An efficient and affordable electrocatalyst are essential for practical energy conversion and storage devices such as fuel cells, water-splitting cells, and metal-air batteries [1, 2]. Atomically dispersed metal catalysts, including single-atom catalysts (SACs) and atomic clusters, have been widely investigated due to their well-defined active sites and high atom utilization efficiency [3, 4]. Transition metal-nitrogen moieties supported on carbon-based materials represent a unique class of atomically dispersed metal catalysts with high electrical conductivity [5]. They are among the most promising candidates to efficiently catalyze a wide range of electrochemical processes, such as hydrogen evolution/oxidation reactions (HER/HOR), CO_2_/CO reduction, oxygen reduction reaction (ORR), and oxygen evolution reaction (OER) [6–9].

In alkaline and acidic electrolytes, Fe–N–C catalysts have been proved the highest ORR performance among the carbon-supported transition metal (Fe, Co, Mn, Cu, etc.) SACs [10]. However, the performance of Fe–N–C catalysts due to the very strong bonding with oxygenated intermediates is still lagging behind the apex of the M–N–C Volcano plot [11, 12]. In addition, the Fe–N–C catalyst suffers activity degradation in an acidic medium due to Fenton reactions. In comparison with the Fe–N–C catalyst, the Co–N–C and Mn–N–C catalysts exhibited better ORR stability in an acidic medium [13, 14]. It has been demonstrated that the ORR performance of Fe–N–C catalysts can be further enhanced by rationally tailoring the geometry and electronic structure of center metal atoms, such as implanting bi-atomic metal cores and substituting the adjacent coordination dopants [15, 16]. Despite tremendous efforts made in the past, the relatively poor durability of Fe–N–C catalysts under highly oxidative environments has held back their practical applications in fuel cells and rechargeable zinc-air batteries (ZABs) [17–19]. Remitting the adverse effects on the properties of catalysts caused by harsh OER working conditions is still a significant challenge for M–N–C catalysts [12, 20]. An efficient multifunctional carbon-based catalyst should be electrochemically stable within a wide working potential window, particularly at high potentials [21, 22]. It has been reported that the oxidation of carbon domains with rich local defects probably leads to a sharp decline of ORR activity [3, 23–25]. Therefore, tracking the dynamic structural evolution of the active triple-phase boundary of M–N–C catalysts is critical to improving their activity and stability.

In this work, we developed a new type of binary-atom catalyst with Fe-N_4_ and Co-N_2_C_2_ structures co-anchored on CNTs. This fine structure can be achieved by precisely controlling the spatial distance between iron and cobalt sites in the trimetallic zeolitic imidazole frameworks. Meanwhile, nitrogen-coordinated Co nanoclusters generated in the carbon matrix further optimize the electronic structure of the Fe-N_4_ active center for efficient ORR. In addition, the degradation mechanisms of catalysts under highly oxidative conditions in alkaline media are revealed by systematic electrochemical studies, combined with microscopy and in-situ X-ray absorption spectroscopy (XAS) analyses. Although in-situ XAS indicated that the M–N–C moieties with graphitized carbon part are stable under the OER process, the corrosion of the amorphous carbon part with Fe-N_4_ active sites leads to the dissolution of the active metal sites and collapse of the mesoporous structure. It will result in a significant decrease in electrochemically active surface area (ESCA) and significantly increased charge- and mass-transport resistances. The highly conductive carbon nanotubes (CNTs) as a robust support keep a high electrochemically active surface area (ESCA) and low charge mass-transport resistances after OER process. Consequently, the electrocatalysis performance of the Fe/Co-CTs/CNTs in PEMFCs and rechargeable ZABs is significantly enhanced.

## Experimental Section

### Synthesis of Catalysts

First, a series of Fe and Co co-doped ZIFs were synthesized by changing the mass ratio. Specifically, the mass ratios of Fe(NO_3_)_3_·9H_2_O: Co(NO_3_)_2_·6H_2_O: Zn(NO_3_)_2_·6H_2_O: 2-mehtylimidazole (2-MIM) were kept at 0.1:0.1:3.39:3.94 (Fe/Co-SAs/NC), 0.5:0.5:3.39:3.94 (Fe/Co-CTs/NC), 1.0:1.0:3.39:3.94 (Fe/Co-NPs/NC). The Fe/Co-FSAs/NC has less atomically dispersed metal active sites than Fe/Co-SAs/NC. In a typical synthetic procedure for the Fe/Co-CTs/NC catalyst, Fe(NO_3_)_3_·9H_2_O (0.5 g), Co(NO_3_)_2_·6H_2_O (0.5 g), and Zn(NO_3_)_2_·6H_2_O (3.39 g) were dissolved in 50 mL methanol to form solution, which was subsequently injected into 350 mL of methanol containing 3.94 g 2-methylimidazole (2-MIM) under ultrasound for 10 min. The mixed solution was heated for 24 h at 60 °C under vigorous stirring. The precipitants were centrifuged and washed with ethanol several times followed by drying in a vacuum at 70 °C overnight. The obtained powder was placed in a tube furnace and heated up to 900 °C for 1 h in N_2_ atmosphere before ammonia activation at 900 °C for 10 min to obtain the final Fe/Co-CTs/NC catalyst. In the synthetic process of precursor for Fe/Co-CTs/CNTs, the CNTs were introduced and encapsulated by Fe- and Co- co-doped ZIF. In a typical synthesis for the catalyst, Fe(NO_3_)_3_·9H_2_O (0.5 g), Co(NO_3_)_2_·6H_2_O (0.5 g), and Zn(NO_3_)_2_·6H_2_O (3.39 g) were dissolved in 50 mL methanol to form a clear solution. CNTs (0.1 g) were dispersed in 350 mL methanol solution containing 3.94 g of 2-methylimidazole (2-MIM) under ultrasound for 10 min. The two solutions were mixed and heated up to 60 °C for 24 h under stirring. The precipitant was centrifuged and washed with ethanol several times and was dried in a vacuum at 70 °C overnight. To this end, the powders were placed in a tube furnace, heated up to 900 °C for 1 h under flowing N_2_ gas, and subjected to ammonia activation for 10 min to obtain the Fe/Co-CTs/CNTs catalyst.

### Physical Characterizations

The morphology of the as-prepared samples was imaged by a scanning electron microscope (SEM, Quanta 450 ESEM, FEI) and a transmission electron microscope (HRTEM, JEM-2100). The surface properties were analyzed by X-ray photoelectron spectroscopy (XPS, VG ESCALAB 220i-XL) equipped with a hemispherical analyzer for a Twin Anode X-ray Source (Al Kα, 1846.6 eV). The crystal structure of the catalysts was analyzed by X-ray diffraction (XRD) (40 kV, 25 mA, Cu Kα radiation, *λ* = 1.5418 Å). The Fe and Co K-edge X-ray absorption near-edge structure (XANES) and Extended X-ray Absorption Fine Structure (EXAFS) data were collected on the 06ID-1 Hard X-ray MicroAnalysis (HXMA) beamline at the Canadian Light Source, a 2.9 GeV third-generation synchrotron source. Data collection configuration used metal Fe and Co foil as energy calibration by in-step calibration for every data set [26].

### Electrochemical Characterizations

The electrocatalytic performance for ORR and eOER reactions was carried out in a standard three-electrode cell (Pine, Model PGSTAT-72637) workstation at room temperature. All electrochemical tests were conducted in either O_2_-saturated or N_2_-saturated KOH electrolyte solution. To subtract the background capacitive current, linear sweep voltammetry was conducted under the same conditions in an N_2_-saturated electrolyte. Catalyst ink was coated on the rotating ring-disk electrode (RRDE, PINE Research Instrumentation) used as the working electrode. A Pt wire and Hg/HgO (KOH, (20%)) electrode were used as the counter and reference electrodes, respectively. Before the electrochemical measurements, the reference electrode was calibrated to a reversible hydrogen electrode (RHE) in an H_2_-saturated electrolyte. The sealed standard three-electrode cell (below) was used for reference electrode calibration. Pt foil was used as both the working electrode and counter electrode, and the electrolyte was saturated with high-purity hydrogen. A rotating ring-disk electrode (RRDE) with a Pt ring and a glassy carbon (GC) disk (5.61 mm diameter) was used as the substrate for the working electrodes. Before use, the GC electrodes in RDE/RRDE were polished using an aqueous alumina suspension on felt polishing pads. The catalyst ink was prepared by mixing 2.0 mg catalyst ultrasonically with 0.4 mL of isopropyl alcohol and 10 mL of 5 wt% Nafion® for more than 30 min. For comparison, commercially available Pt/C (20 wt% Pt) and ruthenium dioxide (RuO_2_, 99%) catalysts were used as the reference.

## Results and Discussion

### Synthesis and Structural Characterization

**Fig. 1:**
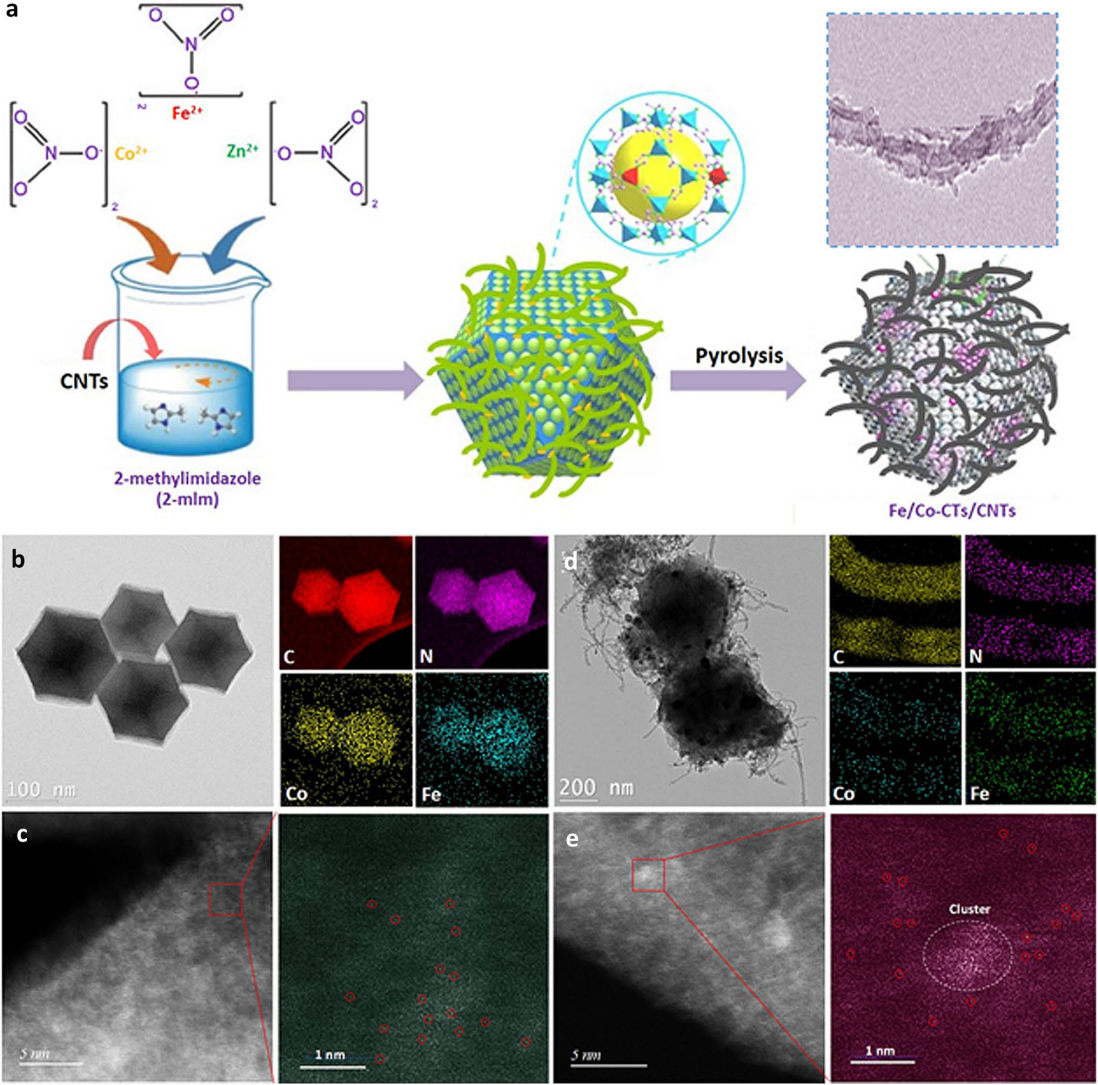
**a** Schematic illustration of the synthesis of hybrids catalyst (Fe/Co-CTs/CNTs). **b** TEM, corresponding element maps showing the distribution of Fe, Co, C, and N. **c** HAADF-STEM image and enlarged image of the Fe/Co-SAs/NC. **d** TEM image of Fe/Co-CTs/CNTs and the corresponding element mappings show the distribution of Fe, Co, C, and N. **e** HAADF-STEM image of CNTs within Fe/Co-CTs/CNTs and enlarged images of CNTs

The Fe/Co-cluster catalyst supported on the carbon nanotubes (Fe/Co-CTs/CNTs) was prepared via two steps (Fig. 1a). In brief, a suspension of CNTs, Fe(NO_3_)_2_, Co(NO_3_)_2_, and 2-mehtylimidazole (2-MIM) with the desired ratio was prepared and heated under stirring to allow the formation of Fe/Co co-doped ZIF-8 (ZnFeCo-ZIFs). Meanwhile, by direct nucleation, growth, anchoring, and encapsulation, highly conductive CNTs were successfully wrapped by Fe/Co co-doped ZIF-8. The hybrid precursor was then annealed at 900 °C in N_2_ atmosphere for 1 h, followed by an ammonia (NH_3_) treatment at 900 °C for 10 min to obtain the final Fe/Co-CTs/CNTs catalyst. For comparison, the metal species supported on the N-doped carbon (NC), such as Fe/Co-single atom catalyst (Fe/Co-SAs/NC), Co-nanocluster decorated Fe/Co-single atom catalyst (Fe/Co-CTs/NC), and Fe/Co-nanoparticle catalyst (Fe/Co-NPs/NC), were prepared by pyrolyzing Fe/Co co-doped ZIF-8 (Fig. S2).

**Fig. 2:**
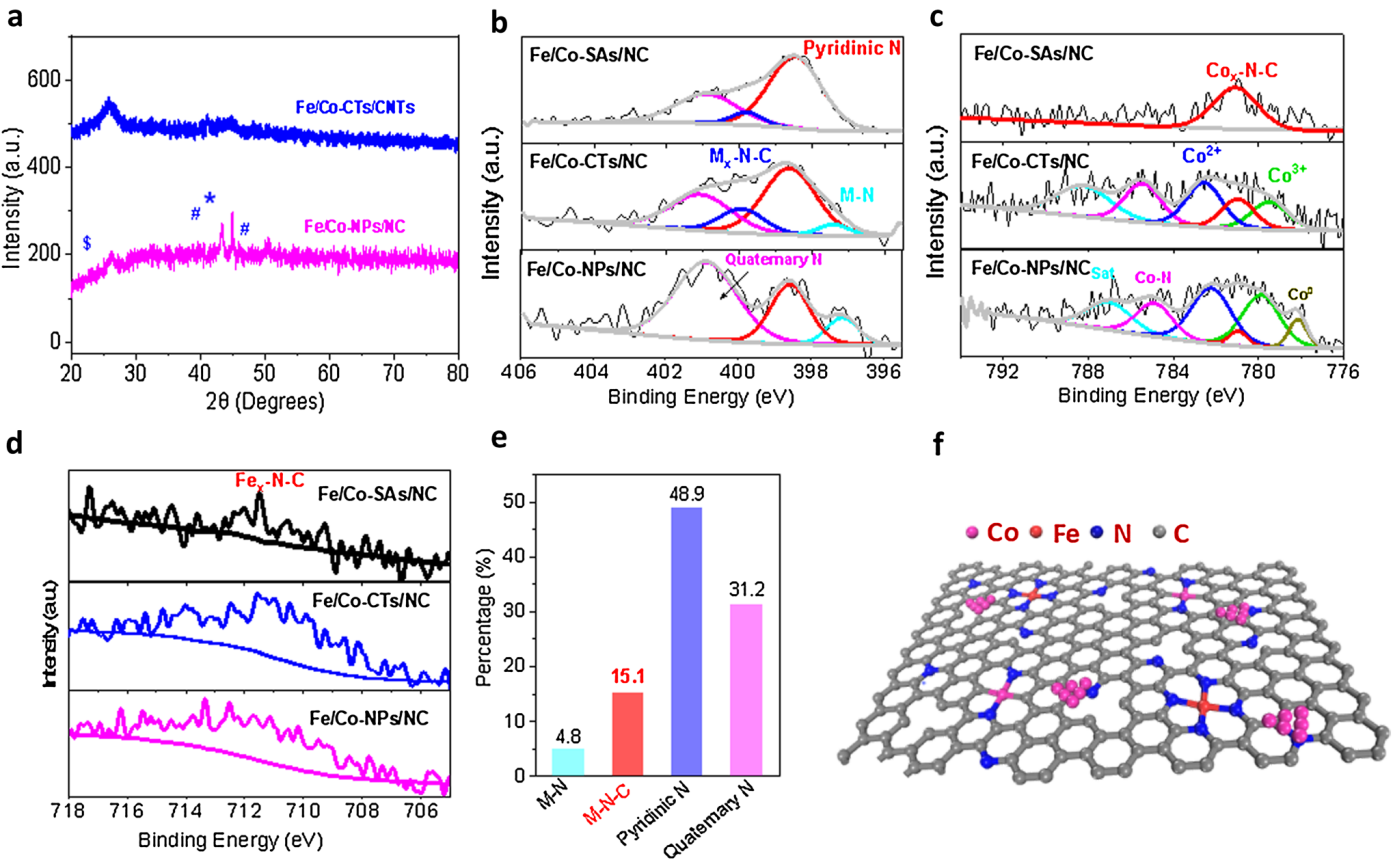
**a** XRD of the Fe/Co-CTs/NC and Fe/Co-NPs/NC. **b–d** High-resolution N 1* s* (**b**), Co 2*p*3/2 (**c**) and Fe 2*p*3/2 (**d**) XPS data of as-prepared samples. **e** The percentage content of four N types relative to total N in the Fe/Co-CTs/NC sample. **f** Schematic model of M–N–C sites decorated by nanoclusters. Co nanoclusters (pink), Fe (red), Co (pink), N (blue), and C (gray)

Figures 1b and S3 show that the Fe/Co-SAs/NC retains its initial dodecahedral shape after thermal treatment; meanwhile, Fe, Co, C, and N are distributed uniformly over the entire architecture of Fe/Co-SAs/NC. Moreover, HAADF-STEM imaging displays many bright spots corresponding to atomically dispersed Fe/Co-atom distributed across the carbon framework in Fe/Co-SAs/NC (Figs. 1c and S3b). In addition to some single atom spots, Fe/Co-CTs/NC and Fe/Co-CTs/CNTs catalysts show nitrogen-coordinated Co nanoclusters and some nanoparticles (Figs. 1d, e and S5, S10, S11). In the case of Fe/Co-NPs/NC catalysts, the small spatial interval between two metal atoms leads to the formation of large metal particles, which results in a sharp decrease in the active site density of atomically dispersed species (Fig. S4) [27]. Even though, the graphitization degree of carbon materials is improved in the presence of the FeCo-based nanoparticles, as reflected by the XRD (Fig. S6). The peak at 26.38° corresponds to the (002) lattice plane of graphite-2H (PDF#41-1487), which is expected to enhance carbon corrosion resistance effectively. In addition, the bimetallic FeCo carbide and alloy in the Fe/Co-NPs/NC are detected (Fig. 2a). The superiority of these active species results in their high durability, excellent recyclability, and OER performance [28–31].

The elemental quantifications for different catalysts, as determined by XPS data, are summarized in Table S1. For the high-resolution N 1*s* spectra (Fig. 2b), the two dominant peaks at 398.5 and 401.0 eV correspond to quaternary- and pyridinic-N, respectively. The N 1*s* XPS peak at 399.9 eV can be attributed to metal-N–C (M–N–C), considered efficient ORR active centers [32, 33]. The Fe/Co-CTs/NC has the highest M–N-C contents (Fig. 2c-d and Table S1), and the percentage of M–N–C increased from 6.7% (Fe/Co-SAs/NC) to 15.1% (Fe/Co-CTs/NC) (Fig. 2e). For the Fe/Co-NPs/NC, the percentage of quaternary-N continuously increased to 60.6 at%; the percentage of pyridinic-N decreased by about 34.2%, indicating that graphitized carbon structures of Fe/Co-NPs/NC with higher corrosion/oxidation resistance lack sufficient defects and nitrogen dopants to host adequate FeN_4_ site densities.

**Fig. 3:**
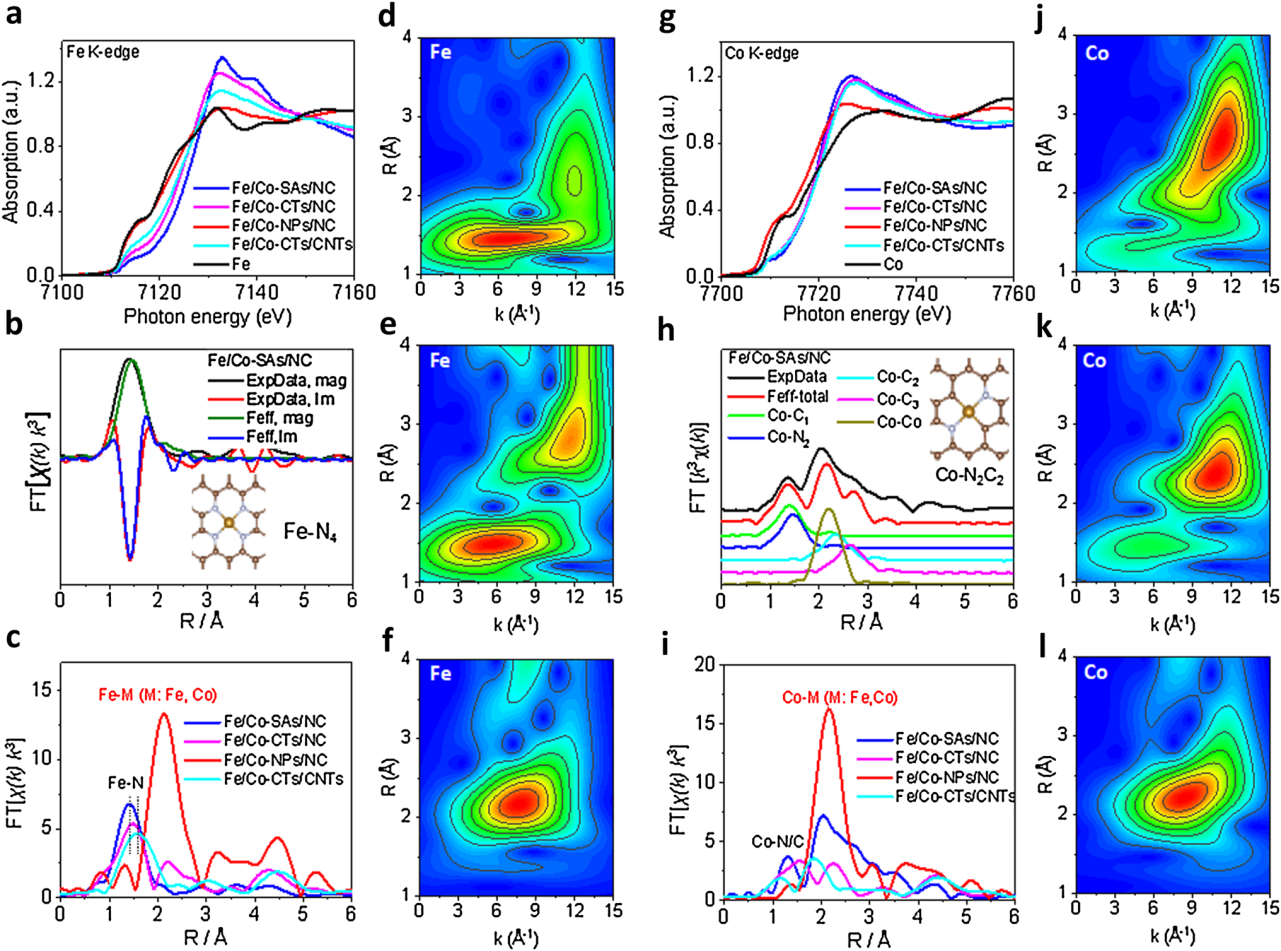
Normalized **a** Fe and **g** Co K-edge XANES spectra of synthesized catalysts and standard samples. Comparison is made for the magnitude and the imaginary part of the Fourier transform of **b** Fe and **h** Co between the experimental and the Feff modeling. The Fourier transforms of **c** Fe and **i** Co K-edge EXAFS oscillations k^3^χ(k) of different catalysts. **d–f** Fe K-edge wavelet transforms (WT)-EXAFS contour plots of **d** Fe/Co-SAs/NC, **e** Fe/Co-CTs/NC, and **f** Fe/Co-NPs/NC. **j-l** Co K-edge WT-EXAFS contour plots of** j** Fe/Co-SAs/NC, **k** Fe/Co-CTs/NC, and **l** Fe/Co-NPs/NC

Homogeneous dispersion of atomic Fe/Co species is confirmed by HR-STEM and XRD analyses in the Fe/Co-SAs/NC sample (as shown in Fig. 1b–e). The atomic-site structures in different catalysts are further investigated by XANES and EXAFS. The results show that the Fe/Co-SAs/NC has the highest Fe and Co oxidation state compared to the Fe_3_O_4_ and CoO references (Figs. 3a, b and S15). The comparison of the first derivative XANES of Fe/Co-SAs/NC with references indicates that the stable valence states for the Fe and Co are + 3 and + 2, respectively [34, 35]. The bonding environment of the Co and Fe atoms is also fitted in R space by Fourier-transformed k^3^-weighted EXAFS (Fig. 3b, h). The Fourier transform curves for Fe/Co-NPs/NC exhibit two peaks at 2.12 and 2.13 Å (Fig. S16), indicating the primary metallic crystal form. In contrast to Fe foil, FePc, Fe/Co-CTs/NC, and Fe/Co-NPs/NC, Fe–Fe/Co bond at 2.46 Å is absent in Fe/Co-SAs/NC, indicating the nature of isolated Fe atoms. Figure 3d–f shows the Fe and Co K-edge wavelet transforms (WT)-EXAFS contour plots for the three k^3^-weighted χ(k) signals based on Morlet wavelets with optimum resolution. The location of the intensity maximum with different coordinates (k, R) is primarily distinguished by path length R and the weight of the coordination atom. The higher the atomic weight of the coordination atom, the higher the maximum intensity in the k-space. As shown in Fig. 3f, l, Fe–Co alloy exhibited an intensity maximum at ~ 7.8 Å^−1^ (Fe–Co contribution). In contrast, for Fe/Co-SAs/NC and Fe/Co-CTs/NC, only a predominant intensity maximum at lower k-spaces (6.3 and 5.7 Å^−1^, respectively) was detected (Fig. 3d, e). The absence of the Fe–Fe/Fe–Co metallic path further certified the nature of isolated Fe atoms in these two samples. Notably, according to the fitting parameters given in Table S2 and the FeN_4_ model, the coordination number of Fe–N within Fe/Co-SAs/NC is four, and the first shell of Fe in the Fe/Co-SAs/NC catalyst can be well-fitted as approximate Fe-N_4_ structure (Fig. 3b). Consistent with EXAFS characterization (Fig. S17 and Table S2), XANES fitting guided by the theoretical DFT modeling support that the Fe bearing in the sample possesses a FeN_4_ type (Figs. S1 and S18–S21). In addition, for the bonding environment of the Co atoms, the fitting results show that Co–C and Co–N bonds are formed and their coordination numbers were all estimated to be ∼2 (Table S4). This result demonstrates that Co–C and Co–N bonds coexist to create active Co–N_2_C_2_ sites jointly. Moreover, the Co Fourier transform curve of Fe/Co-SAs/NC showed an additional peak at 2.6 Å (Fig. 3h), close to the Co–Co contribution of Co foil. The coordination number of Co–Co within Fe/Co–SAs/NC is ∼1 (Table S4), suggesting the existence of nitrogen-coordinated Co clusters along with the Co–C and Co–N bonds in the Fe/Co-SAs/NC catalyst. For the Fe/Co-CTs/NC and Fe/Co-CTs/CNTs, the Co Fourier transform curves combined with WT-EXAFS contour plots showed the coexistence of Co–Co bonding and Co–N/C bonding (Fig. 3i–l). The intense interaction between clusters and M_*x*_–N sites can favor ORR by activating the O–O bond, thus facilitating a direct 4 e^−^ process [36].

### Electrochemically-Measured Catalytic Activity in Acidic Media

**Fig. 4:**
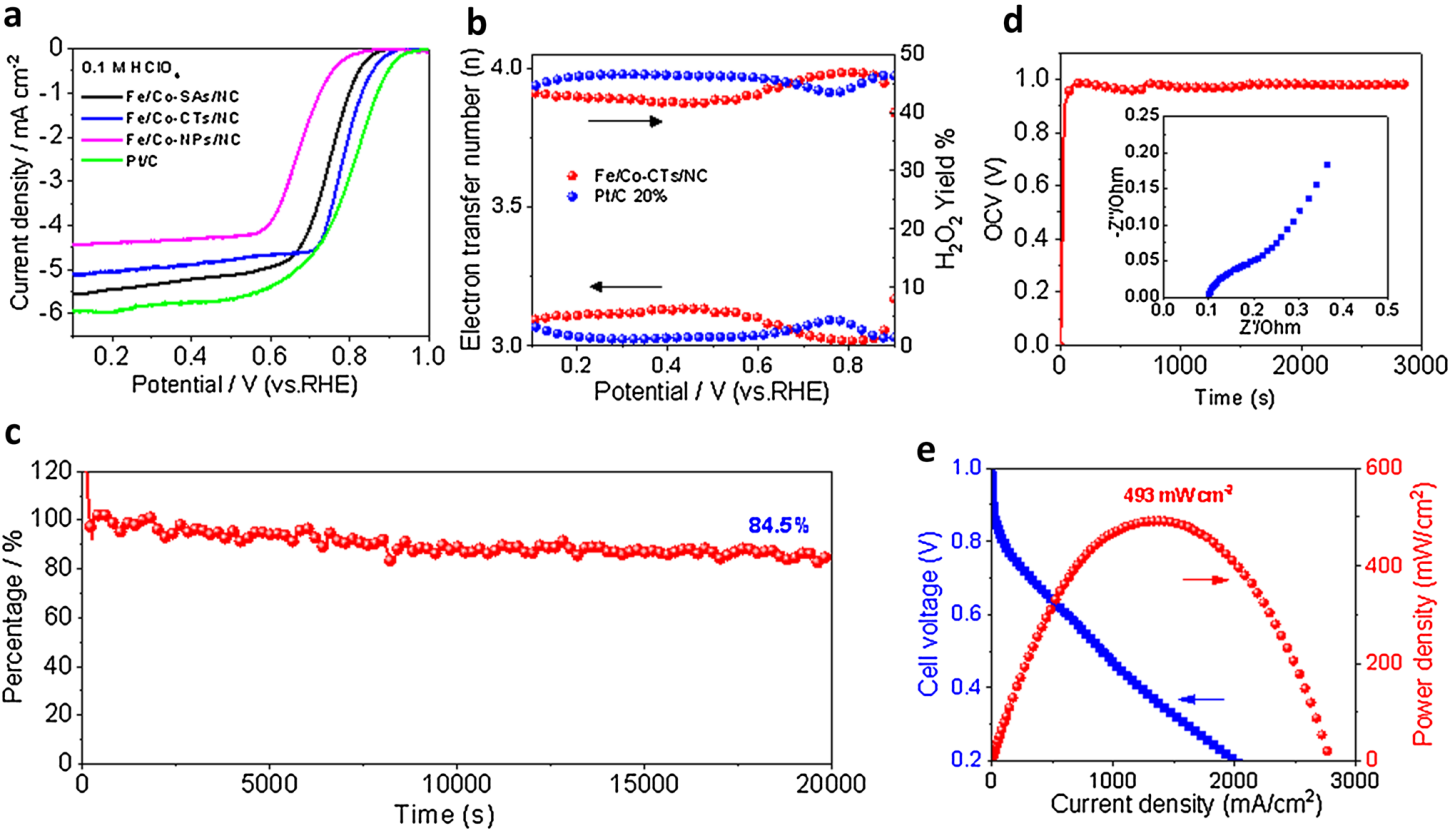
**a** ORR polarization plots of as-prepared samples (rotation rate: 1600 rpm) in O_2_-saturated 0.1 M HClO_4_ at a scan rate of 5 mV s^−1^. **b** Percentages of H_2_O_2_ produced and the electron transfer numbers of Fe/Co-CTs/NC and Pt/C catalysts. **c** Chronoamperometric responses of Fe/Co-CTs/NC at 0.7 V in an O_2_-saturated 0.1 M HClO_4_ solution. **d** Electrochemical impedance spectra (EIS) at an open-circuit voltage (OCV) of H_2_/O_2_ fuel cell measured at 80 °C using the as-prepared Fe/Co-CTs/NC. **e** The electrochemical performance (*I-V* curves) of the H_2_/O_2_ fuel cell was measured at 80 °C and 1.0 bar back pressure

The ORR activity and selectivity (H_2_O_2_ yield) of all catalyst samples were evaluated by using a rotating ring-disk electrode (RRDE) in the 0.1 M HClO_4_ electrolyte (Fig. 4a). The Fe/Co-CTs/NC with the highest percentage of M–N–C active sites shows high ORR activity in acidic media as reflected by the distinct ORR peak at 0.7 V versus the reversible hydrogen electrode (RHE) (Fig. S22). Therefore, increasing the percentage of the M–N–C active sites is an effective method to generate high ORR activity in an acidic electrolyte. Compared to Fe/Co-SAs/NC and Fe/Co-NPs/NC catalysts, Fe/Co-CTs/NC showed the highest activity with an onset potential (*E*_onset_) of 0.90 V and an *E*_1/2_ of 0.79 V. The H_2_O_2_ yield of Fe/Co-CTs/NC catalyst remained below 6.4% at all potentials, and even dropped to 0.78% at *E*_1/2_ (0.79 V), corresponding to a high electron-transfer number of 3.98, which suggests a near 4e^−^ oxygen reduction pathway (Fig. 4b). The ORR activity of Fe/Co-CTs/NC in this work is competitive with the state-of-the-art Pt-free ORR catalysts in an acid electrolyte (Table S5) and close to the traditional Pt/C [37, 38]. In addition, Fe/Co-CTs/NC exhibits remarkable durability with a high persisted current of 84.5% after 20,000 s under potentiostatic operation at 0.7 V (Fig. 4c). To exclude the impact of Co-based nanoclusters, Fe/Co-CTs/NC was leached into the 0.5 M H_2_SO_4_ solution for 24 h at 80 °C. After acid leaching, unstable metallic species were removed. The atomically dispersed metallic clusters and M–N–C atomic sites supported on the NC via the strong chemical M–N/C interaction were left (Fig. S23a, b). Compared with initial Fe/Co-CTs/NC, acid-leached Fe/Co-CTs/NC showed a loss of 12 mV in *E*_1/2_, indicating that the strong interaction between isolated Fe/Co–N–C sites and adjacent Co nanoclusters can further enhance ORR performance (Fig. S23c). It suggests that the optimal regulation of the electronic and geometric structure of isolated Fe/Co–N–C sites can be achieved by the coexistence of Co nanoclusters.

The Fe/Co-CTs/NC catalyst was further evaluated in membrane electrode assemblies (MEAs) for the PEMFCs. The MEA cell exhibits a small ohmic resistance (0.098 Ω) and high open-circuit voltage (0.98 V) using H_2_ and O_2_ as fuel and oxidant, respectively, suggesting a high intrinsic ORR activity in a practical fuel cell environment (Fig. 4d). The Fe/Co-CTs/NC cathode can generate current densities of 0.62 and 2.01 A cm^−2^ at 0.6 and 0.2 V, respectively (Fig. 4e). The corresponding peak power density is up to 493 mW cm^−2^. Fe/Co-CTs/CNTs showed relatively lower power density (385.5 mW cm^−2^) due to the low concentration of M–N–C active sites per unit mass; however, such a power density has a much higher M–N–C utilization efficiency (increased by about 50%). The CNTs further enhance the intrinsic mass transport and internal ohmic resistance of the MEAs (Figs. S24 and S25).

### Electrochemical Catalytic Activity in Alkaline Media

**Fig. 5:**
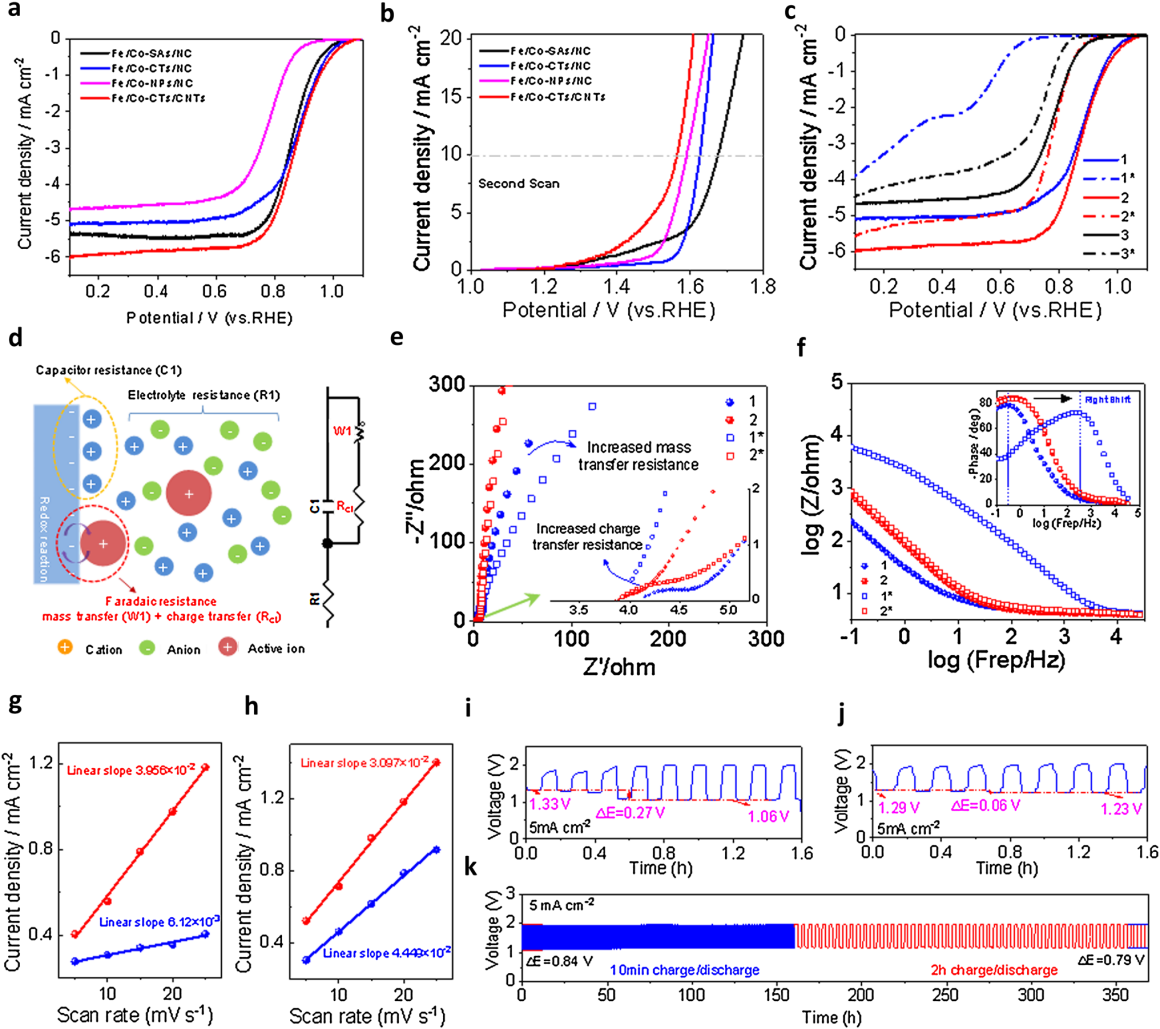
**a** ORR polarization plots of as-prepared samples (rotation rate: 1600 rpm) in 0.1 M KOH at a scan rate of 5 mV s^−1^. **b** OER polarization plots (second OER scan) of as-prepared samples in 1.0 M KOH at a scan rate of 5 mV s^−1^. **c** ORR polarization plots of as-prepared samples (rotation rate: 1600 rpm) after the OER process in 0.1 M KOH at a scan rate of 5 mV s^−1^. **d** Schematic of a catalyst-loaded electrode in contact with the liquid electrolyte, and the corresponding simulated equivalent circuit.** e** Electrochemical impedance spectra (EIS) at a three-electrode system. **f** Bode plots of EIS spectra. Fe/Co-CTs/NC (named 1), Fe/Co-CTs/CNTs (named 2), Fe/Co-NPs/NC (named 3), and * denote the corresponding sample after the first OER process. The capacitive current of **g** Fe/Co-CTs/NC and h) Fe/Co-CTs/CNTs measured at 1.15 V versus RHE as a function of scan rate. **i–k** Galvanostatic discharge and charge cycling stability of **i** Fe/Co-CTs/NC and **j, k** Fe/Co-CTs/CNTs. Electrolyte: 6.0 M KOH with 0.2 M zinc acetate

As shown in Figs. 5a and S31, the Fe/Co-NPs/NC decorated with only metal nanoparticles exhibited much lower ORR activity than the other catalysts (Fe/Co-SAs/NC, Fe/Co-CTs/NC, and Fe/Co-CTs/CNTs). The best ORR activity was achieved by the Fe/Co-CTs/CNTs in terms of the onset (*E*_onset_) and half-wave (*E*_1/2_) potentials. The *E*_1/2_ of Fe/Co-CTs/CNTs (0.873 V) is almost the same as that of Pt/C (0.878 V vs. RHE), outperforming the most non-precious-metal catalysts reported so far (Table S6). The OER catalytic activity of all catalyst samples was investigated in a 1.0 M KOH electrolyte. Figure S27a shows their first scan of linear sweep voltammetry (LSV) from 1.0 to 2.0 V. The OER activity follows the order of Fe/Co-CTs/NC > Fe/Co-SAs/NC > Fe/Co-CTs/CNTs > Fe/Co-NPs/NC. However, the increased current response in the range of 1.2–1.7 V may be partly attributed to the redox reaction of Co^2+^/Co^3+^ and carbon corrosion for the Co-based catalysts [39, 40]. As shown in Fig. S27a, the OER performance of Fe/Co-SAs/NC and Fe/Co-CTs/NC drops off precipitously at about 1.7 V, which may be due to the occurrence of a phase transformation. In addition, the CV curves of Fe/Co-SAs/NC and Fe/Co-CTs/NC become stable after one initial cycle (Figs. S28a and S29b), suggesting irreversible phase transformation in the first cycle. It can be due to the dissolution of the heteroatoms N and the formation of high-valence oxoanions during OER [41, 42]. Consequently, Fe/Co-CTs/CNTs catalyst shows an *E*_j=10_ of 1.56 V which is smaller than those of Fe/Co-NPs/NC (1.59 V), Fe/Co-CTs/NC (1.63 mV) and Fe/Co-SAs/NC (1.67 V) (Figs. 5b and S27b). There was very little change in overpotential (*η*_a_) of the Fe/Co-CTs/CNTs and Fe/Co-NPs/NC (Fig. S28) after exposure to a strong oxidation condition. The results convey that the introduction of CNTs with rich atomically dispersed Fe/Co-atom active sites can significantly improve antioxidant capability. Moreover, after 2500 cycles of ADTs in the potential range of 1.4–1.8 V, the Fe/Co-CTs/CNTs catalyst demonstrated excellent stability with only 21 mV increased potential at 50 mA cm^−2^ (Fig. S29). In addition, the acid-leaching treatment led to a significant decline in OER activity, particularly at a high potential range (increased 100 mV at 50 mA cm^−2^) (Fig. S30). Therefore, the OER activity of Fe/Co-CTs/CNTs catalyst at high potentials can largely be linked to the presence of the Fe/Co nanoparticles or clusters rather than single M–N–C active sites.

### Effect of Harsh OER Conditions on ORR Performance

To reveal the influence of the high potential operation (OER) on the ORR activity, we further evaluated the ORR catalytic activities in the 0.1 M KOH. Meanwhile, the single ORR catalytic activities of the catalysts without the OER process were also investigated to analyze the influence of the OER process [43]. The ORR activities follow an order of Fe/Co-CTs/CNTs > Fe/Co-NPs/NC > Fe/Co-CTs/NC > Fe/Co-SAs/NC after the OER process. Moreover, after 5000 cycles of ADTs in the range of 0.6 to 1.1 V in O_2_-saturated 0.1 M KOH, the Fe/Co-CTs/CNTs catalyst demonstrated excellent stability with a loss of only 15 mV in *E*_1/2_ (Fig. S32). The bifunctional ORR and OER activity parameters (∆*E*) for Fe/Co-CTs/CNTs in 1.0 M KOH is as low as 0.588 V (Fig. S33), which is much smaller than that of most non-precious-metal catalysts reported so far (Table S2).

After the strong oxidation condition of the OER process, the Fe/Co-CTs/CNTs catalyst demonstrated high retained ORR activity (*E*_1/2_ = 0.78 V, after the OER process) (Figs. 5c and S34). For comparison, the Fe/Co-CTs/NC catalyst after the OER process loses more than 300 mV in its *E*_1/2_ for ORR (0.88 and 0.57 V, before and after the OER process, respectively). The degradation of Fe/Co-NPs/NC is relatively small, indicating that the negative effect of carbon oxidation is low due to the improved degree of graphitization. However, Fe/Co-NPs/NC with limited active sites still exhibits much lower ORR performance (*E*_1/2_ = 0.74 V, after the OER process) than Fe/Co-CTs/CNTs (0.78 V). Consequently, the bifunctional activity parameter (∆*E*) after strong oxidation is as low as 0.78 V for the Fe/Co-CTs/CNTs, which implies that nearly 81% of the bifunctional catalytic activities can be restored. Therefore, the introduction of CNTs as the carbon substrate can significantly improve the bifunctional catalytic activities and stability. The optimized chemical composition consisting of stable atomically dispersed FeCo clusters on the support can still provide sufficient electroactive sites for electrocatalytic reactions after the OER process. As indicated by the CVs in a proper potential range without redox processes and the corresponding capacitive current plots against the scan rate (Figs. S35 and 5 g, h), the Fe/Co-CTs/CNTs sample after the OER process still has the largest double-layer capacitance (*C*_dl_) (Fig. 5h). It suggests that the large electrochemically active surface area (ESCA) retained ensures excellent bifunctional activities and stability.

To characterize the kinetics of the electrode reactions before and after the OER process, the impedance response EIS analyses were performed to identify the active triple-phase boundary. The catalyst-loaded electrode in contact with the liquid electrolyte and the corresponding equivalent circuit is illustrated in Fig. 5d. Charge transfer resistance (*R*_ct_) and mass transfer resistance (W1) represent the Faradaic resistance, which is directly related to the electrocatalytic activity of the catalysts. Compared to Fe/Co-CTs/NC, Fe/Co-CTs/CNTs exhibits a smaller Faradaic charge-transfer resistance and mass-transfer resistance reflected by the smaller semicircle and higher slope of mass transfer region (Figs. 5e and S36a). For the Fe/Co-CTs/NC catalyst after the OER process, the prominent peak shift to the high-frequency region and the significantly increased diameter of the semicircle reveals an increased *R*_ct_ and W1. The increase of the electrochemical impedance (*Z*) in the high-frequency region is related to the remarkably higher *R*_ct_ and W1 (Figs. 5f and S36b). Therefore, the strong oxidation condition can lead to the collapse of mesoporous structure and dissolution of heteroatom dopants, significantly reducing the exposed active sites and increasing *R*_ct_ and W1. For the Fe/Co-CTs/CNTs after the OER process, the small semicircle and the nearly unchanged mass transfer region indicate that the *R*_ct_ and W1 after a strong oxidation condition are still much lower than Fe/Co-CTs/NC after the OER process. Therefore, atomically dispersed Fe/Co-atom active sites distributed on highly conductive CNTs can effectively resist the increase of Faradaic resistance due to carbon corrosion.

### Rechargeable Zn-Air Batteries in Two- and Three-Electrode Configuration

To avoid the ORR activity loss problem of Fe/Co-CTs/NC catalyst upon exposure to the positive oxidation potential of OER, we designed a unique rechargeable ZAB device with a tri-electrode configuration. As shown in Fig. S39a, OER and ORR electrocatalysts were loaded onto the two separate electrodes for charge and discharge, which could be optimized individually. The initial charge–discharge voltage gap (*E*_gap_) at 20 mA cm^−2^ was only 0.56 V (Fig. S39b), which is much smaller than that of previously reported (0.7 V) [44]. After operating at 5.0 mA cm^−2^ for a total of 8 h (Fig. S39c), the battery's performance remains nearly unchanged. Although the tri-electrode configuration provides high cycling stability and the flexibility of catalyst preparation, it inevitably increases their volume and weight, ending with reduced volumetric energy. The outstanding bifunctional catalytic activity and high oxidation resistance of Fe/Co-CTs/CNTs make it a promising air electrode material in a two-electrode ZAB (Fig. S40a). For comparison, Fe/Co-CTs/NC and Fe/Co-CTs/CNTs were first studied in a two-electrode ZAB with 1.0 M KOH. The battery discharge performance of Fe/Co-CTs/NC deteriorated noticeably after several cycles (Fig. 5i). Due to ORR activity loss, the discharge overpotential was 270 mV larger than the first cycle. As expected, the ZAB with Fe/Co-CTs/CNTs exhibited considerable oxidation resistance when charged and discharged galvanostatically at 5.0 mA cm^−2^ (Fig. S5j). Particularly, after cycling operation for 370 h at 5.0 mA cm^−2^, the voltage gap remained nearly unchanged (0.79 V), indicating the superior stability of Fe/Co-CTs/CNTs in the two-electrode ZAB (Fig. 5k).

### Dynamic Study of Atomic Metal-N–C Active Sites Under Harsh OER Conditions

**Fig. 6:**
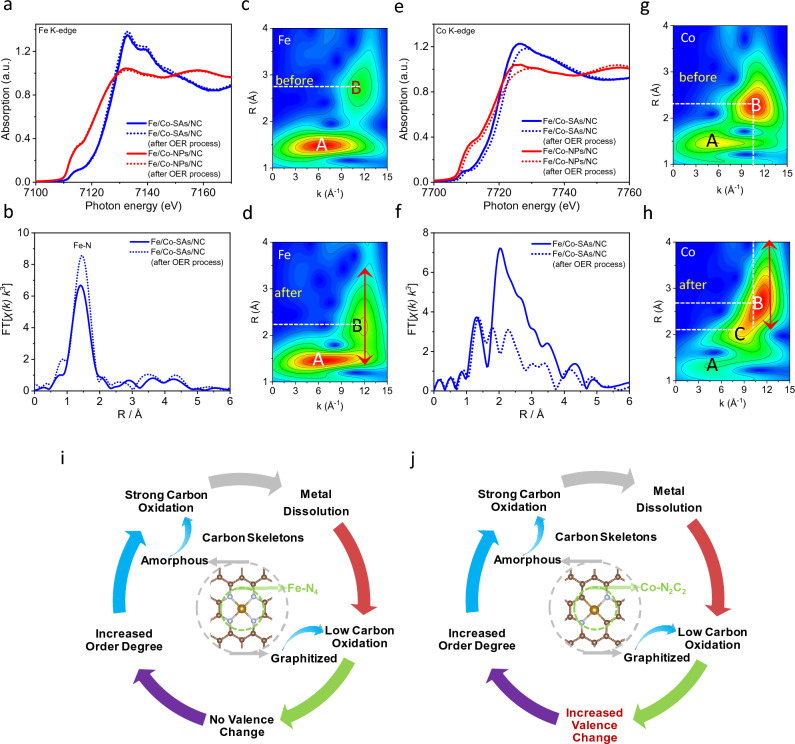
**a, e** Normalized Fe and Co K-edge XANES spectra of Fe/Co-SAs/NC and Fe/Co-NPs/NC before and after OER process. The corresponding Fourier transforms of **b** Fe and **f** Co K-edge EXAFS oscillations k^3^χ(k) (k-weight: 3). **c, d** Fe and **g, h** Co K-edge WT-EXAFS contour plots of Fe/Co-SAs/NC before and after OER process. **i, j** Schematic of the impacts of OER process on the atomically dispersed Fe and Co active centers

To investigate the changes in the coordination environment and chemical state of the absorbing center after high oxidation potentials, XAFS measurements were also performed. As shown in Fig. 6a, the Fe K-edge XANES spectra of the Fe/Co-SAs/NC and Fe/Co-NPs/NC nearly overlap after the high oxidation potentials test, and there is no apparent shift to higher energy values. It indicates that the Fe valence states in a metallic crystal form and atomically dispersed Fe^3+^ form both remain unchanged. Moreover, Fe K-edge WT-EXAFS of Fe/Co-SAs/NC before and after OER process both show a predominant intensity maximum at 6.3 Å^−1^ suggesting the unchanged FeN_4_ configure (Fig. 6c, d). The impact of OER on the Fe/Co-SAs/NC is “quantitative”, and not “qualitative”, i.e., the nature of the Fe–N–C sites has not been changed throughout OER. For the Fe in the single atom form within the Fe/Co-SAs/NC, the order degree of FeN_4_ configuration was enhanced after OER process, demonstrated by significantly decreasing the DW parameters by 68% and 31% for paths Fe–N and Fe–C, respectively (Table S3). The FeN_4_ sites with amorphous carbon skeleton could result in high plane distortion and tend to dissolve under high oxidation potentials. Moreover, the Fe–N bond length without expansion was detected after the OER tests (Fig. 6b–d, and Table S3), which also indicates the unchanged oxidation state of Fe in the Fe/Co-SAs/NC [45]. The obvious extension of B intensity loop along path length R suggests that the high oxidation potential would affect the structure of the outer carbon shell due to the amorphous carbon oxidation (Fig. 6d, h). In addition, the Fe in a metallic crystal form in the Fe/Co-NPs/NC is also not significantly impacted by the high oxidation potentials (Fig. S43a, b). In contrast, the Co XAFS spectra of the Fe/Co-SAs/NC and Fe/Co-NPs/NC both show a rigid positive shift to higher energy after OER tests, suggesting that the Co in a metallic nanoparticles and atomically dispersed form are all oxidized to a higher valence state (Fig. 6e) [46, 47]. However, the negative shifts of the maximum intensities with an amplitude of about 0.4 Å^−1^ suggest the replacement of O– with Co-coordination on Fe/Co-NPs/NC (Fig. S43c, d). In addition, the high oxidation potential process compacts the overall framework of the Co local structural environment in the Fe/Co-SAs/NC (Fig. 6f). Particularly, high oxidation potential compels the outer shell carbon coordination surrounding the Co-N_2_C_2_ configuration, which is supported by the reduced CN for paths Co–C_2_ and Co–C_3_ (Table S4). The location of intensity maximum B of Fe/Co-SAs/NC before and after OER process is primarily distinguished by path length R. The apparent extension of B intensity loop along path length R also suggests that the high oxidation potential would further enhance the disordering of the outer carbon shell (Fig. 6g, h). In conclusion, as shown in Fig. 6i, j, the structural effect can potentially detach unstable M–N–C sites from the carbon substrate to form metal oxides, dramatically reducing the content of M–N–C active sites [48]. In addition, high oxidation potentials will result in the collapse of the amorphous carbon matrix surrounding the M–N–C active sites, which is a major factor in the large overpotentials for the ORR/OER.

## Conclusion

We have successfully synthesized isolated binary-atom Co/Fe sites (Fe-N_4_ and Co-N_2_C_2_) with nitrogen-coordinated cobalt nanoclusters anchored on CNTs by adjusting the spatial distance between the iron and cobalt sites in a MOF structure. The coexistence of Fe-N_4_, Co-N_2_C_2_, and cobalt nanoclusters has been identified, resulting in high ORR activity in both alkaline and acid media. The antioxidative CNTs effectively stabilize the atomically dispersed Fe/Co-atom active sites, enabling high ESCA and low charge- and mass-transport resistances. As a result, the Fe/Co-CTs/CNTs exhibit enhanced bifunctional oxygen electrocatalysis stability and high cycling stability in rechargeable ZABs. Moreover, our electrochemical measurements and various characterization techniques, including in-situ EXAFS, XPS, and EIS analysis, comprehensively reveal the degradation mechanisms of catalysts under highly oxidative conditions in alkaline media. Our findings demonstrate that the electronic structure of the Fe-N_4_ active center can be optimized with adjacent Co-N_2_C_2_ sites and nitrogen-coordinated cobalt nanoclusters, leading to improved ORR and OER performances. This study provides an effective strategy for designing efficient multifunctional electrocatalysts with potential applications in PEMFCs and rechargeable zinc-air batteries.

### Supplementary Information

Below is the link to the electronic supplementary material.

Supplementary file1 (PDF 7281 kb)
