# Supplementary material for: Engineering Fe-N4 Electronic Structure with Adjacent Co-N2C2 and Co Nanoclusters on Carbon Nanotubes for Efficient Oxygen Electrocatalysis
Source: Nanomicro Lett. 2023 Oct 20;15:232. doi: 10.1007/s40820-023-01195-2 (PMC10589168; doi:10.1007/s40820-023-01195-2)
Supplement: Supplementary file 1 — Supplementary file1 (PDF 7281 kb) [file 40820_2023_1195_MOESM1_ESM.pdf]

Supporting Information for

Engineering Fe-N_4 Electronic Structure with Adjacent Co-N_2C_2 and Co Nanoclusters on Carbon Nanotubes for Efficient Oxygen Electrocatalysis

Mingjie Wu[1, 2, 3], Xiaohua Yang[4], Xun Cui[1], Ning Chen[5], Lei Du[2], Mohamed Cherif[2], Fu-Kuo Chiang[6], Yuren Wen[7], Amir Hassanpour[2], François Vidal[2], Sasha Omanovic[3], Yingkui Yang[1, *], Shuhui Sun[2, *], Gaixia Zhang[4, *]

[1] State Key Laboratory of New Textile Materials and Advanced Processing Technologies, Wuhan Textile University, Wuhan 430200, P. R. China

[2] Institut National de la Recherche Scientifique (INRS), Center Énergie Matériaux Télécommunications, Varennes, Québec J3X 1P7, Canada

[3] Department of Chemical Engineering, McGill University, 3610 University Street, Montreal, QC H3A 0C5, Canada

[4] Department of Electrical Engineering, École de Technologie Supérieure (ÉTS), Montréal, QC, H3C 1K3, Canada

[5] Canadian Light Source (CLS), 44 Innovation Boulevard, Saskatoon, SK S7N 2V3, Canada

[6] National Institute of Low-Carbon-and-Clean-Energy, 102211 Beijing, P. R. China

[7] School of Materials Science and Engineering, University of Science and Technology Beijing, 100083 Beijing, P. R.
China

*Corresponding authors. E-mail: ykyang@wtu.edu.cn (Y. Yang); shuhui.sun@inrs.ca (S. Sun); gaixia.zhang@etsmtl.ca (G. Zhang)

S1 XAS Characterizations

The X-ray absorption spectroscopy (XAS) measurements were performed at the Canadian Light Source (CLS) located at the University of Saskatchewan, a 2.9 GeV third-generation synchrotron source. The Fe and Co K-edge X-ray absorption near-edge structure (XANES) and Extended X-ray Absorption Fine Structure (EXAFS) were collected on the 06ID-1 Hard X-ray MicroAnalysis (HXMA) beamline. The experiment was performed in fluorescence mode using a Canberra 32 Ge germanium array detector.[1] In the experiment, a Si(111) monochromator crystal and Rh mirrors (collimating and focusing mirrors) were used during data collection. The beamline monochromator was detuned to 50% of its full flux to reduce the impact of the X-ray beam's high harmonic components; the detune energy point was set at the end of the XAFS data collection energy range at 13.0 $Å^{-1}$. The monochromator energy was first calibrated at the Co K-edge using Fe and Co reference foils from the EXAFS Materials Inc.. The same reference foil was arranged between the ionization chamber detector I1 and I2 throughout the Co experiment. Therefore, the in-step energy calibration is available for each scan. 100% helium gas was used in all three ionization chamber detectors during the experiment. The scan step-sizes used were 10 eV/step, 0.2 eV/step, and 0.05 $Å^{-1}$/step, respectively, for the pre-edge from -200 to 30 eV, XANES from -30 to 40 eV, and XAFS regions from 40 eV to 12 $Å^{-1}$ of the data range.[2]

Analyses of both the near edge (on an energy scale) and extended range (in the R space) XAS spectra were performed using Athena software.

S2 Liquid Zinc-air Battery Assembly

The zinc-air batteries were tested in home-built electrochemical cells; the electrolyte was 1.0 or 6.0 M KOH with a 0.2 M zinc acetate solution. The Liquid rechargeable zinc-air battery was assembled with current collectors (nickel foam for cathode), a polished zinc plate, and a catalyst layer (CL) coated gas diffusion electrode (GDE). To prepare the air electrode, a definite volume of homogeneous catalyst ink consisting of Fe/Co-CTs/CNTs catalyst, Nafion solution (5 wt.%), and isopropanol was dropped onto a gas diffusion layer (GDL) (loading: 2 mg cm^{-2}) with an exposed active area of 1.13 cm^2. Then Ni foam, GDE, and hydrophobic and breathable membrane were pressed together to form the integrated air electrode. For comparison, batteries using Fe/Co-CTs/NC and 20 wt.% Pt/C catalysts were fabricated by the same method. Polarization data were collected using a galvanodynamic method at a scan rate of 1.0 mA s^{-1}.

S3 Fuel Cell Test

Catalyst ink was prepared using the following procedure: 10 mg of the catalyst was mixed with 272 mL of a 5% Nafion solution, 206 mL of ethanol, and 147 mL of deionized (DI) water. The mixture underwent sonication for 15 minutes, followed by agitation for another 15 minutes. Then, 284 µL of this ink was dispensed onto a 1.14 cm^2 carbon paper (Sigracet 25BC from Ion Power) and dried at 80°C for 1 hour to form the Gas Diffusion Electrode (GDE) for the cathode. The ratio of Nafion to the catalyst was maintained at 1.25. The Membrane Electrode Assembly (MEA) was fabricated by sandwiching a 211 membrane between the cathode GDE and a commercially sourced Pt/C anode GDE. During testing, the flow rates of H_2 and O_2 were both maintained at 0.3 Normal Liters Per Minute (NLPM) with a backpressure of 0.5 bar, and the cell temperature was held at 80 °C.

S4 Calculation

Tafel plot calculation and electron transfer number calculation were based on the previously reported method.[1, 3] ECSA was calculated using ECSA= $C_{dl}/(40*m_{loading})$, where $m_{loading}$ is the loading mass of the catalyst per geometrical area of the electrode. The C_{dl} (double-layer capacitance) was determined by measuring the capacitive current associated with double-layer charging from the scan-rate dependence of cyclic voltammetric stripping. For this, the potential window of cyclic voltammetric stripping was about 1.1 V to 1.19 V versus RHE (1.0 M KOH solution). The scan rates were 5 mV s^{-1}, 10 mV s^{-1}, 15 mV s^{-1}, 20 mV s^{-1} and 25 mV s^{-1}. The C_{dl} was estimated by plotting the j_a at 0.59 V (where j_a are the c anodic current densities) versus RHE against the scan rate, in which the slope was the C_{dl}.

S5 Computational Methods

All the first-principles DFT calculations in this work were performed without symmetry restrictions using the Vienna ab initio simulation package (VASP) code. For these calculations, we used the parametrization of Perdew-Burke-Ernzerhof of the exchange-correlation functional within the generalized gradient approximation (GGA) formalism. No dispersion correction has been included in the calculation as it should be negligible in such compact systems involving covalent bonds. The energy cut-off was set to 400 eV while the Brillouin zone was sampled on 4×4×4 Gamma-Pack k-point grid. An initial graphene sheet, represented by a periodic plate with cell dimensions of a = 12.74 Å and b = 15.00 Å, was used to model the carbon support. A 20 Å thick void layer was included in the z-direction

to minimize possible artificial interactions between the periodic sheets. A number of carbon atoms were removed from the slab and replaced by FeN_x and/or CoN_x moieties. All atomic positions in the supercell were fully relaxed without any constraints using the conjugate gradient algorithm, and the total energy convergence criterion was set to 10^{-6} eV. We have optimized four structures, as illustrated in Fig. S1.

S6 Supplementary Figures and Tables

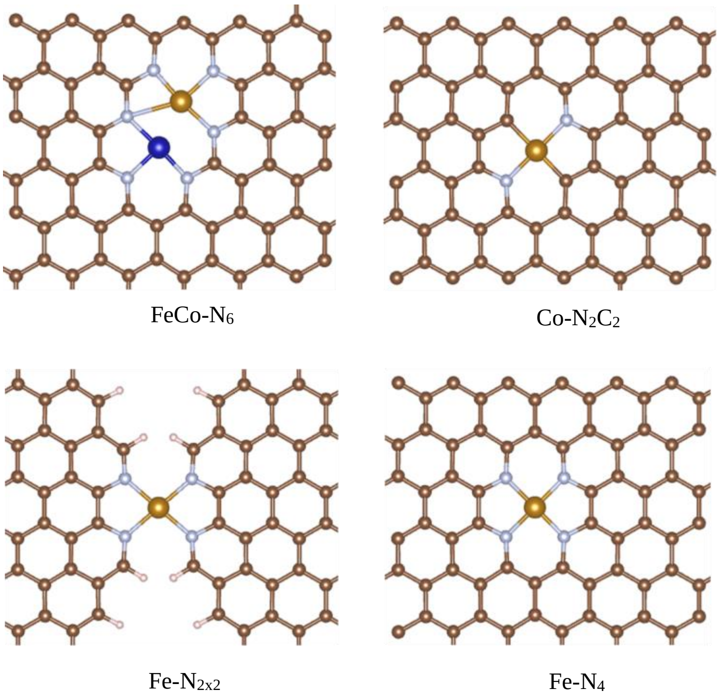

Fig. S1 Four DFT-optimized structures used in conjunction with the XAS characterizations. Brown: carbon, white: nitrogen, yellow: iron, blue: cobalt. In the Fe-N_{2x2} structure, bonds of carbon atoms are completed with hydrogen

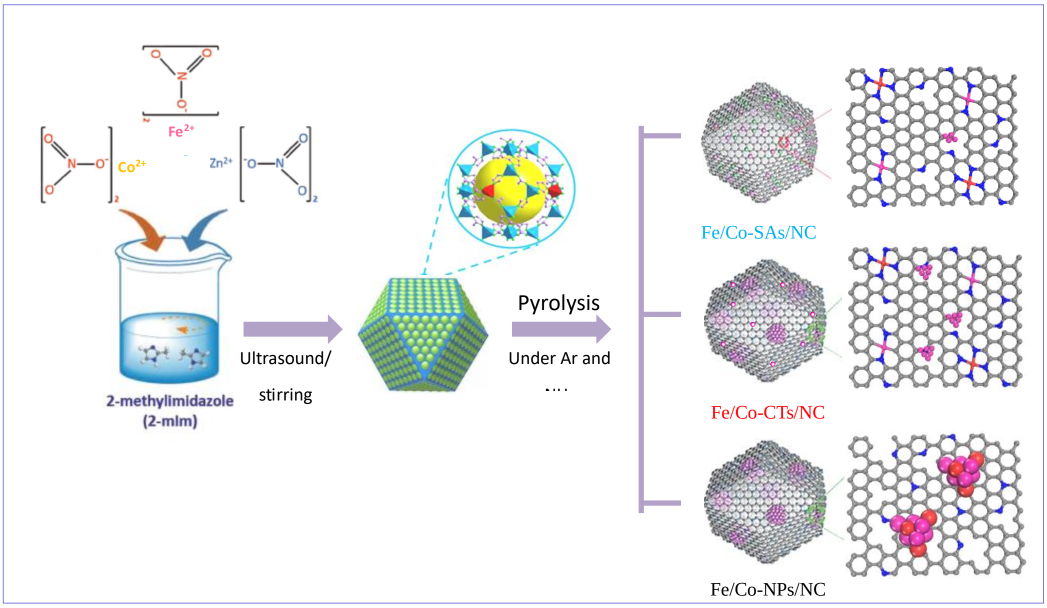

Fig. S2 Schematic illustration of the synthesis of Fe/Co-SAs/NC, Fe/Co-CTs/NC, and Fe/Co-NPs/NC. The mass ratio among $Fe(NO_3)_3{\cdot}9H_2O$, $Co(NO_3)_2{\cdot}6H_2O$,

$Zn(NO_3)_2 \cdot 6H_2O$, and 2-mehtylimidazole (2-MIM) was kept at 0.1:0.1:3.39:3.94 (Fe/Co-SAs/NC), 0.5:0.5:3.39:3.94 (Fe/Co-CTs/NC), 1.0:1.0:3.39:3.94 (Fe/Co- NPs/NC). The Fe/Co-FSAs/NC own less metal active sites than Fe/Co-SAs/NC

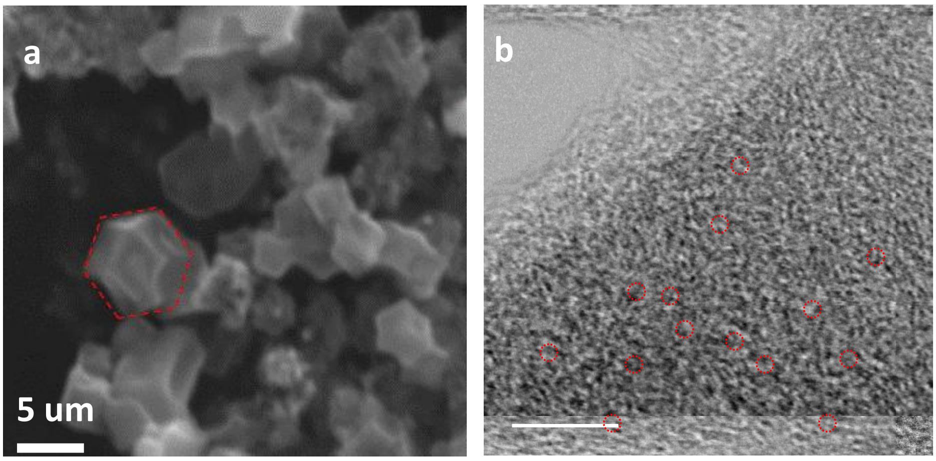

Fig. S3 a) SEM images and **b**) HRTEM image of Fe/Co-SAs/NC

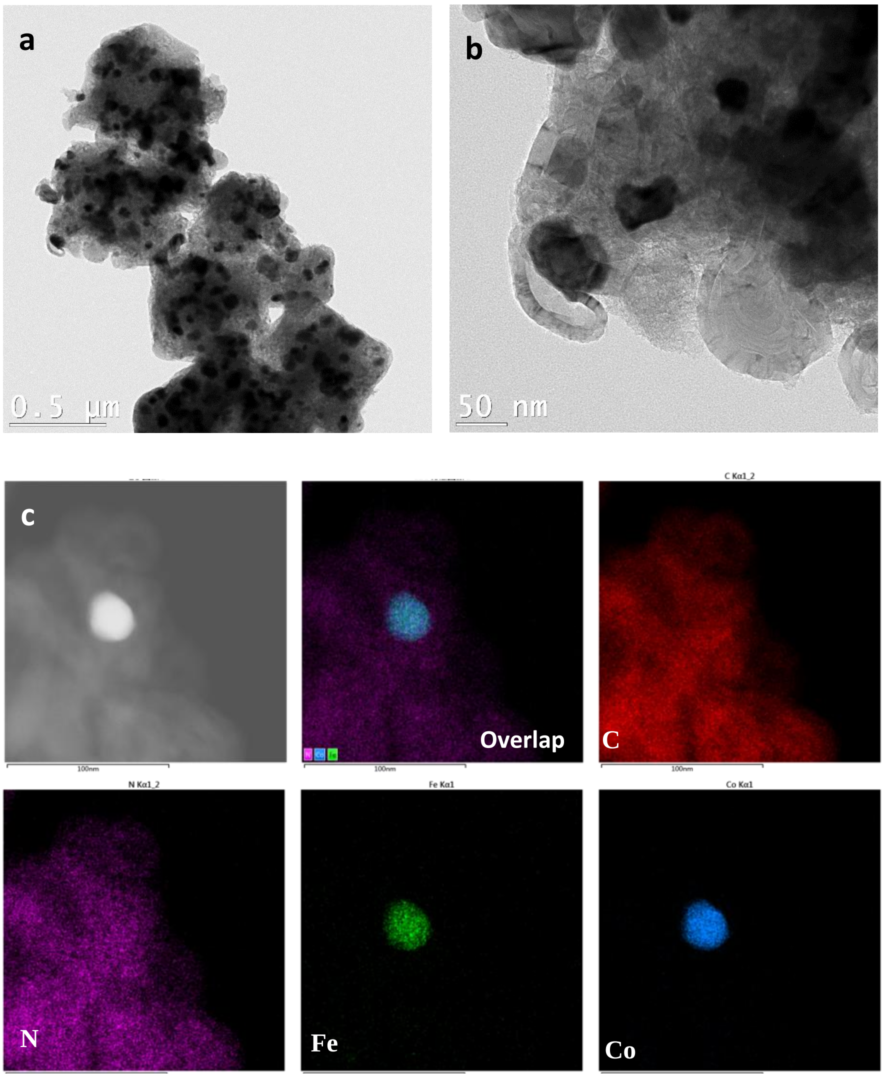

Fig. S4 a, b) TEM images of Fe/Co-NPs/NC. **c**) HAADF-STEM image, the corresponding element maps showing the distribution of Fe, Co, C, and N

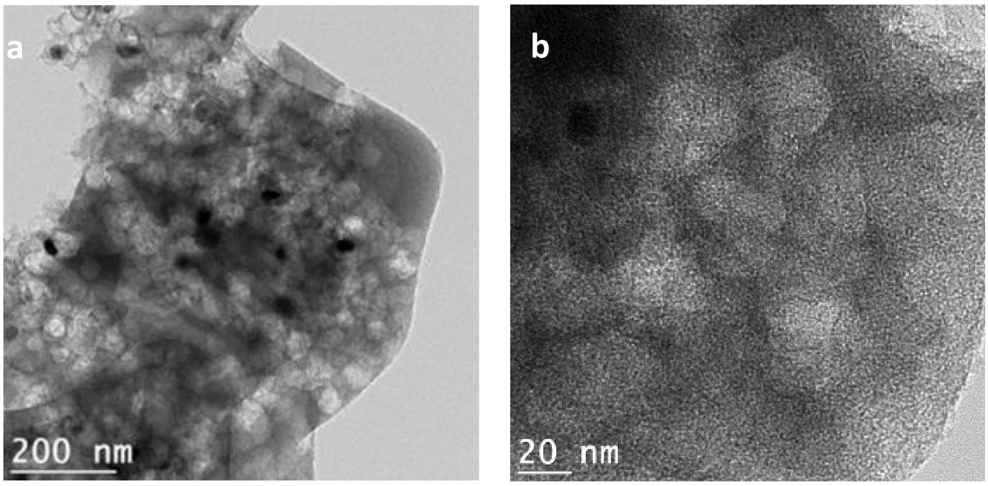

Fig. S5 a-b) TEM images of Fe/Co-CTs/NC

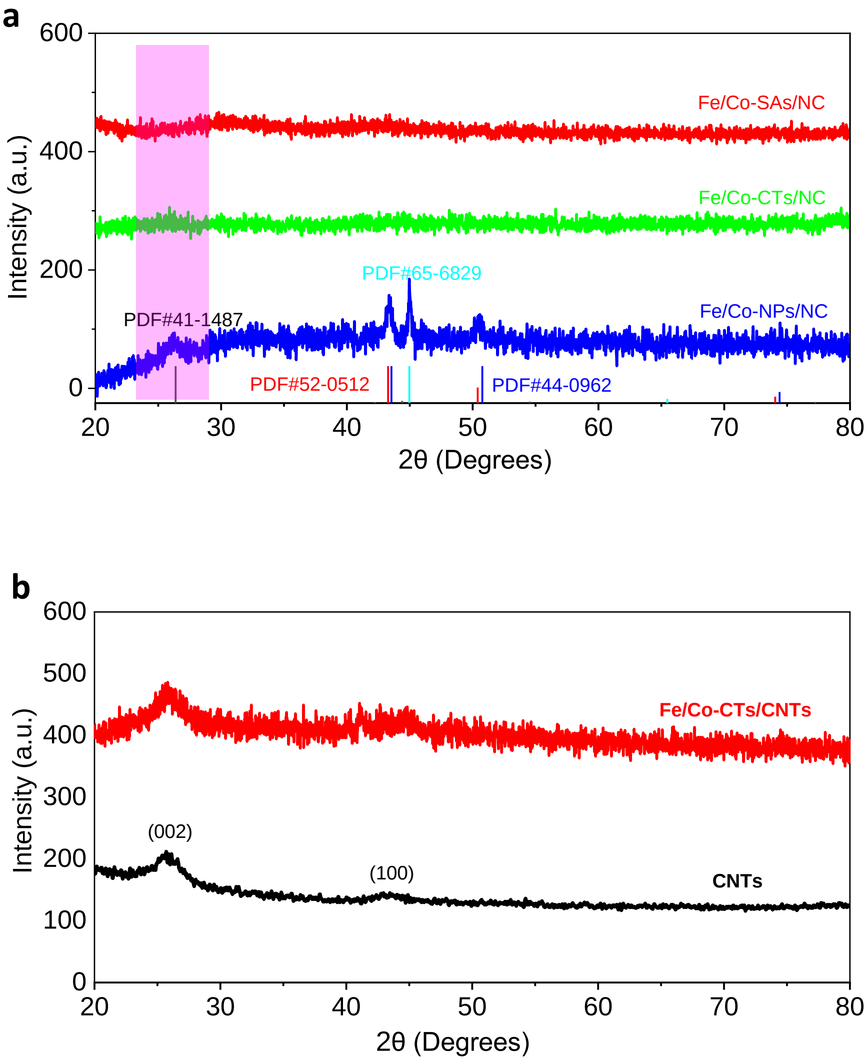

Fig. S6 a, b) The XRD of the as-prepared samples

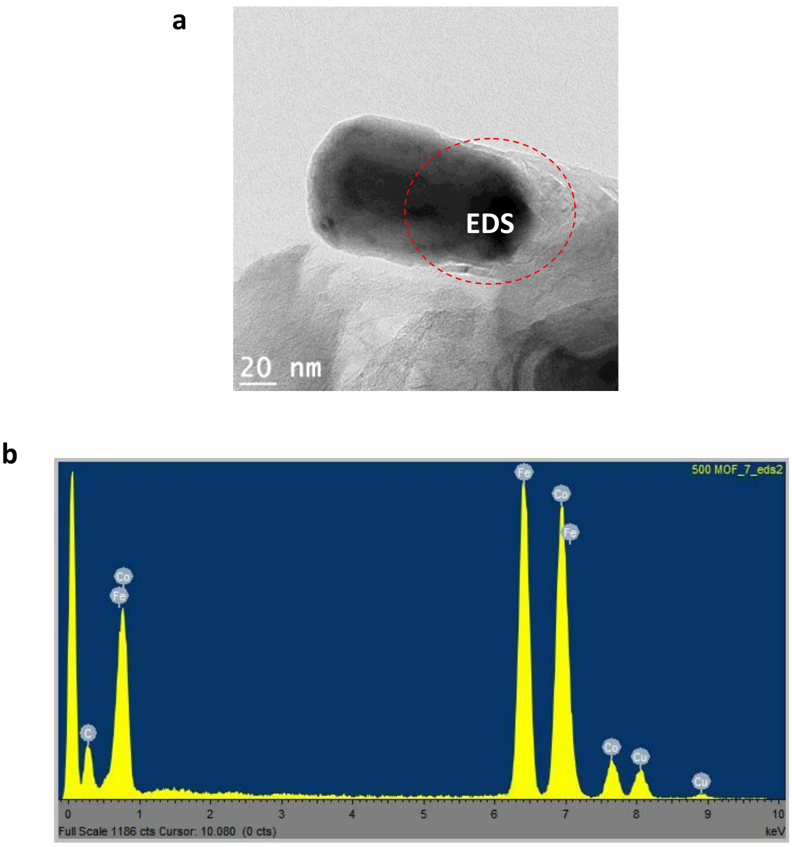

Fig. S7 a) TEM images of Fe/Co-NPs/NC. **b**) EDS elemental analysis of the selected area

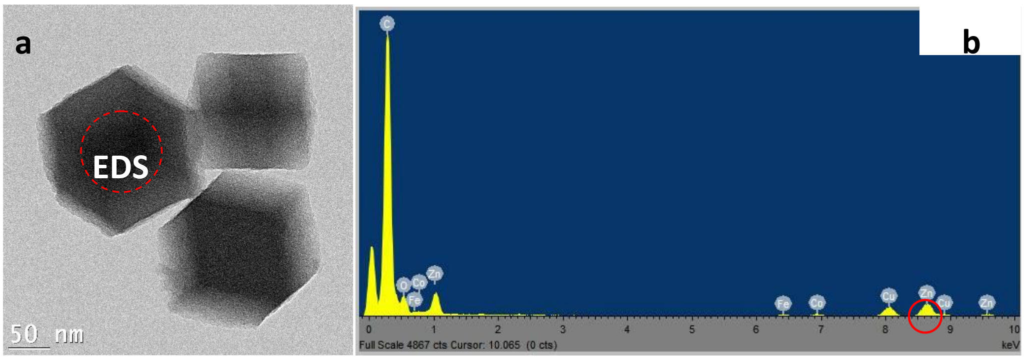

Fig. S8 a) TEM images of Fe/Co-SAs/NC. **b**) EDS elemental analysis of Fe/Co-SAs/NC

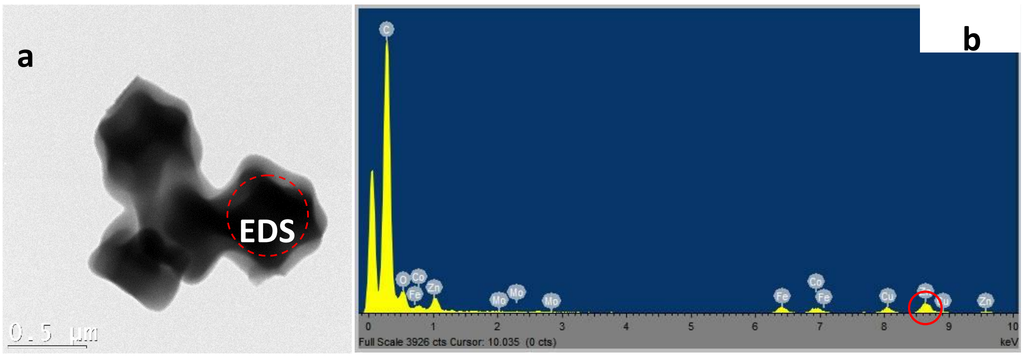

Fig. S9 a) TEM images of Fe/Co-CTs/NC, respectively. **b**) EDS elemental analysis of Fe/Co-CTs/NC

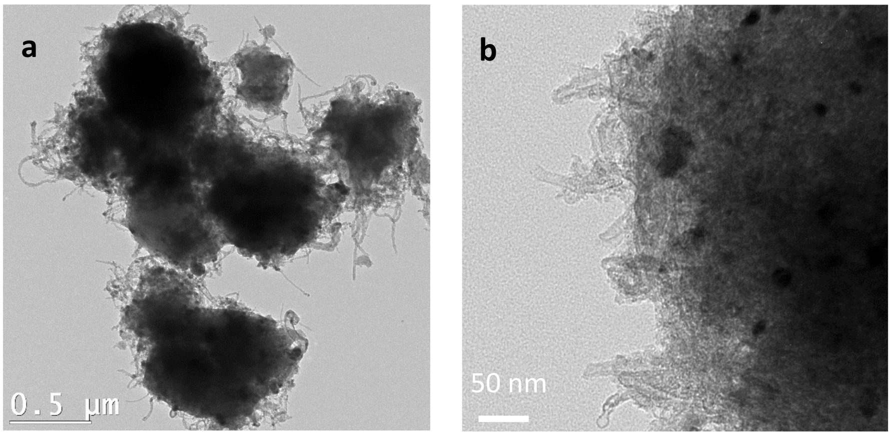

Fig. S10 a, b) TEM images of Fe/Co-CTs/CNTs

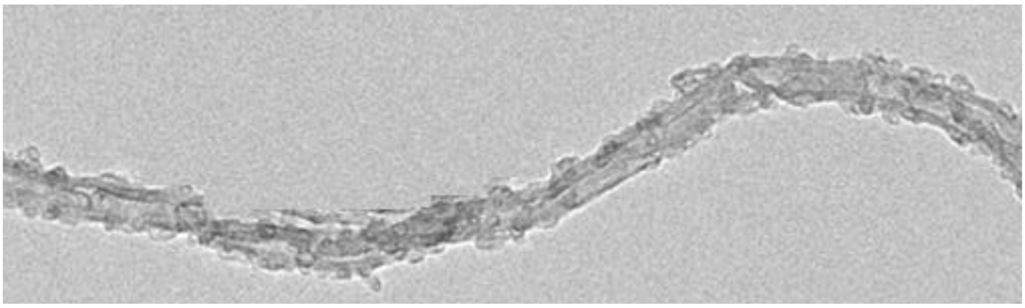

Fig. S11 TEM image of CNTs within Fe/Co-CTs/CNTs

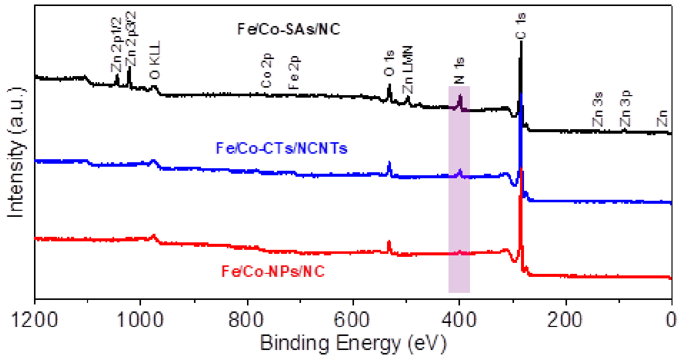

Fig. S12 XPS survey of the as-prepared samples

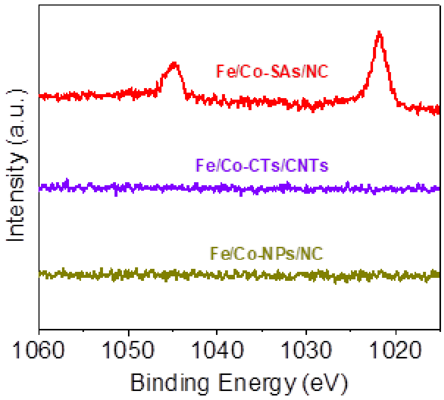

Fig. S13 The high-resolution Zn 2p spectra of the as-prepared samples. It shows that the introduction of CNTs and metal nanoparticles is conducive to removing Zn atoms

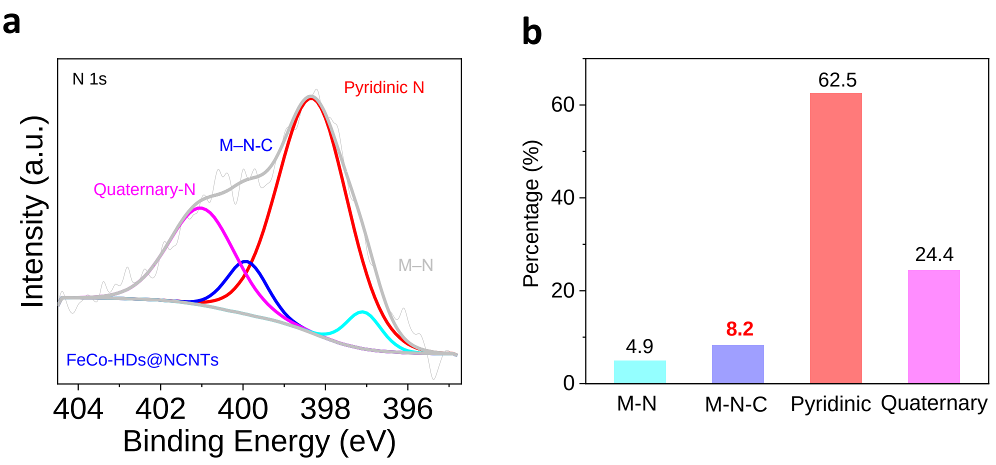

Fig. S14 a) The high-resolution N 1s spectra of the Fe/Co-CTs/CNTs. **b**) The percentage content of four N types relative to total N in the Fe/Co-CTs/CNTs sample

Table S1 Percentage content of four N types relative to total N

Types	Quaternary-N (%)	Pyridinic-N (%)	M–N-C (%)	M-N (%)
Fe/Co-SAs/NC	30.3	63.0	6.7	0
Fe/Co-CTs/NC	31.2	48.9	15.1	4.8
Fe/Co-NPs/NC	60.6	28.8	0	10.6
Fe/Co-CTs/CNTs	24.4	62.5	8.2	4.9

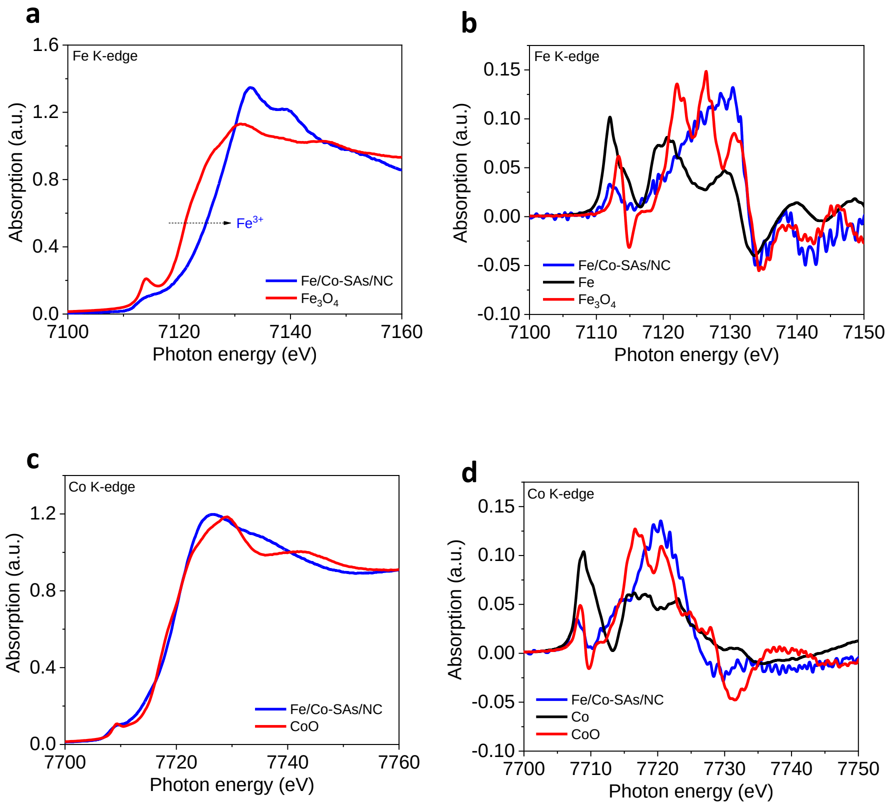

Fig. S15 a, c) Normalized Fe and Co K-edge XANES spectra of various catalysts. **b, d**) The corresponding first derivatives of XANES spectra

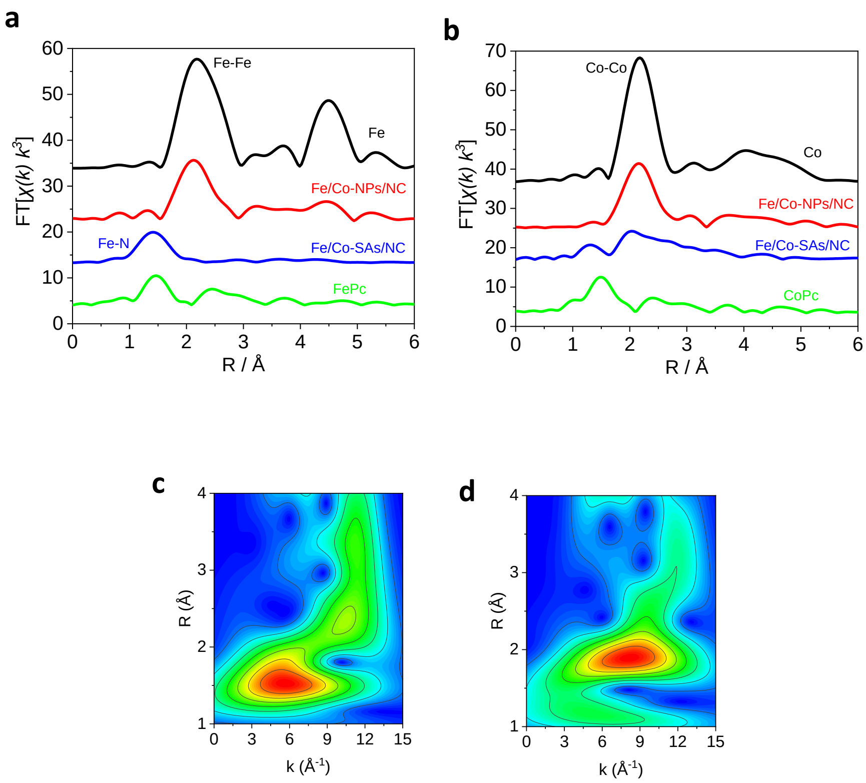

Fig. S16 The Fourier transforms of **a-b**) Fe and **c-d**) Co K-edge EXAFS oscillations $k^3\chi(k)$ of Fe/Co-SAs/NC, Fe/Co-NPs/NC and standard samples, including FePc (Iron(II) phthalocyanine) and CoPc (Cobalt(II) phthalocyanine). **c**) Fe and **d**) Co K-edge WT-EXAFS contour plots of Fe/Co-CTs/CNTs

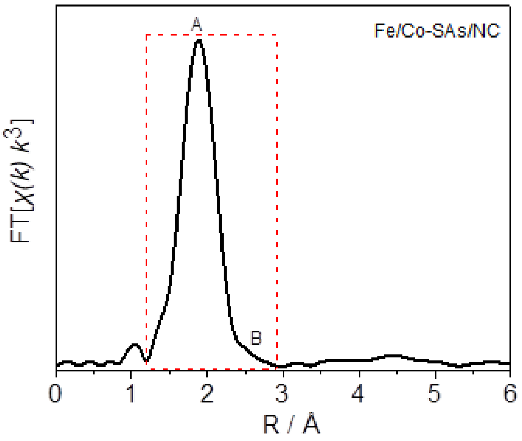

Fig. S17 Fe XAFS data from Fe/Co-SAs/NC: EXAFS in the magnitude of Fourier transform for $k^3\chi(k)$ with nearest neighbor features "A" and "B" indicated

EXAFS characterization was guided by the theoretical DFT modeling and focused on the nearest neighbor Fe local structural environment. The latter is attributed by EXAFS to the experimentally resolved first shell Fourier transform (FT) peak with its major feature peaked as "A" at around 1.41 Å and a shoulder feature "B", carried on the high R wing side of the feature "A". Guided by the DFT models FeCo-N_6, Fe-N_2C_2, Fe-$N_{2\times2}$, and Fe-N_4 (see Fig. S1), the corresponding Fe local structural environment was derived and used for theoretical amplitudes and phases scattering calculation by using the software Feff 7.02 (Rehr and Albers, 2000), respectively, guiding the followed EXAFS R space curve fitting.

The XAFS data reduction and the followed R space curve fitting were performed using ATHENA software (Ravel and Newville, 2005) and WINXAS (version 2.3, Ressler, 1997), respectively. The first inflection point of the Fe K edge XANES was defined as the experimental E_0, and the post-absorption edge background was estimated by the cubic spline fit. The Gaussian window function was used for Fourier transform over *k* data range of 2.5-10.7 Å^{-1} for $k^3\chi(k)$ with a window parameter of 30%. The R space curve fitting was performed for R data window 0.8-2.4Å.

Table S2 DFT model vs. R space curve fitting result of Fe/Co-SAs/NC.

DFT ID	DFT model			R space curve fitting				
	Path	CN	R	CN	R	DW	E0	Fitting residua
$FeCo\text{-}N_6$	$Fe\text{-}N_1$	1	1.84	1.4	1.92	0.0010[a]	1	29.6
	$Fe\text{-}N_2$	2	1.97	2.3	2.00	0.0010[a]		
	Fe-Co	1	2.26	0.3	2.26	0.0064		
	$Fe\text{-}N_3$	1	2.48	0.8	2.45	0.0070		
$Fe\text{-}N_2C_2$	Fe-C	2	1.88	2.2	2.04	0.0010[a]	-6	19.0
	Fe-N	2	1.90	2.2	1.92	0.0016		
	Fe-C	4	2.66	4.2	2.57	0.0076		
$Fe\text{-}N_{2x2}$	Fe-N	4	2.03	4.2	1.96	0.0038	-6	21.6
	Fe-C	4	2.87	0.5	2.90	0.0038		
$Fe\text{-}N_4$	Fe-N	4	1.87	4.0	1.95	0.0034	-6	17.3
	Fe-C	4	2.65	4.0	2.53	0.0058		

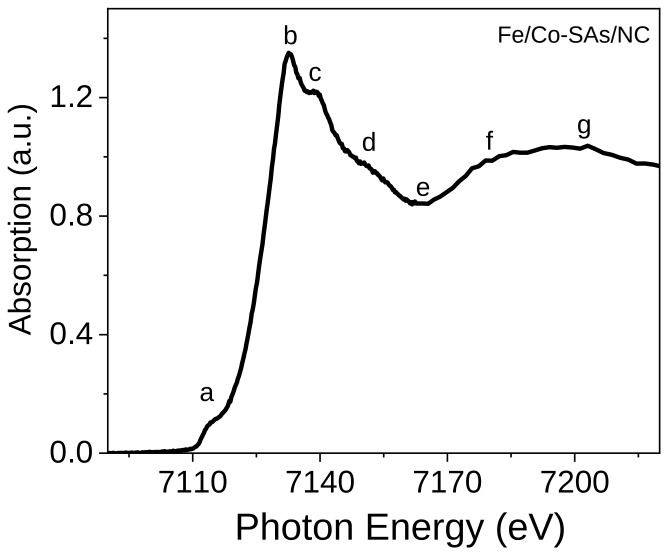

Fig. S18 Fe XAFS data from Fe/Co-SA/NC: XANES with features “a” to “g” labeled

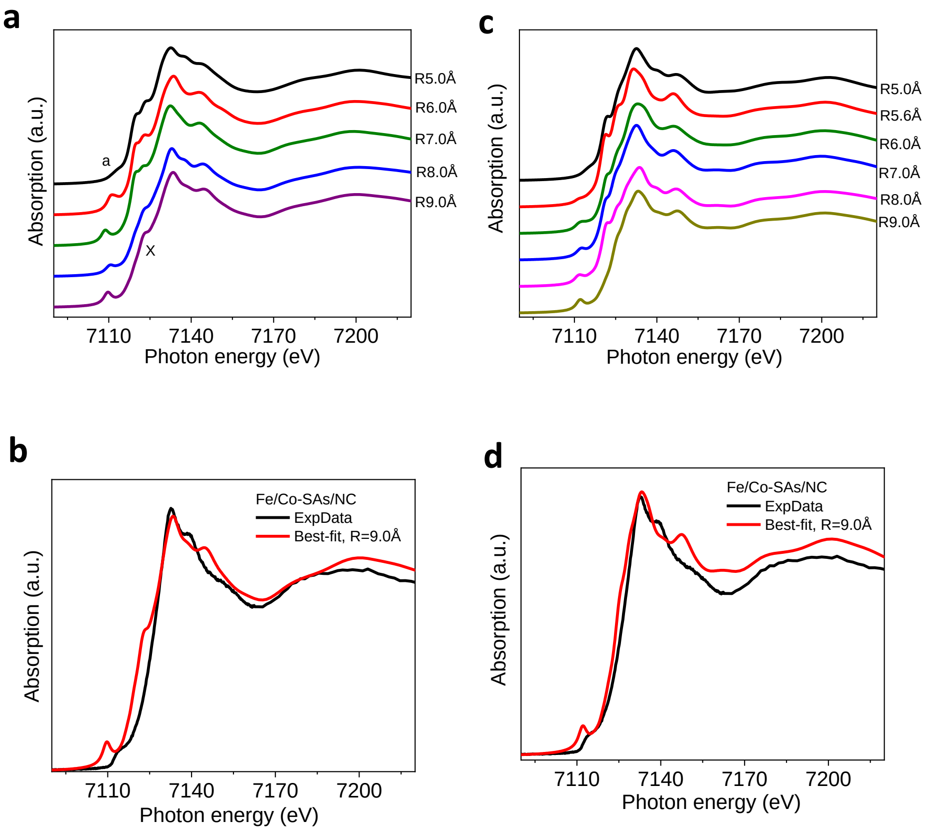

Fig. S19 DFT-based theoretical XANES systems. **a**) XANES system form the first cycle of modeling. **b**) The XANES best fit of the first cycle. **c**) XANES system form the second cycle modeling based on the adjusted structural system guided by EXAFS result. **d**) The XANES best fit of the second cycle. XANES modeling was performed in two cycles

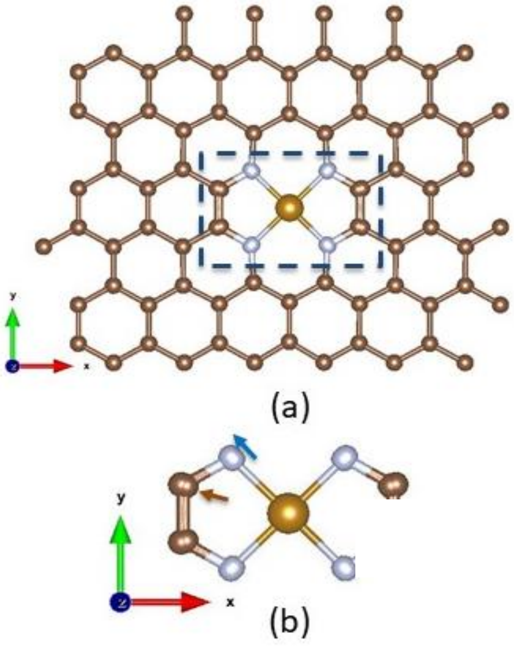

Fig. S20 a) The 9Å cluster from the DFT Fe-N_4 model. b) The Fe nearest neighbor coordination through bonding from N_4 up to C_4

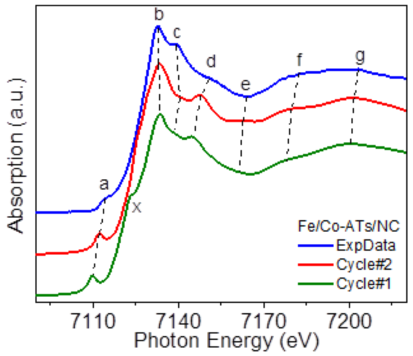

Fig. S21 Comparison between the experimental and the XANES modeling best fit of two cycles of modeling

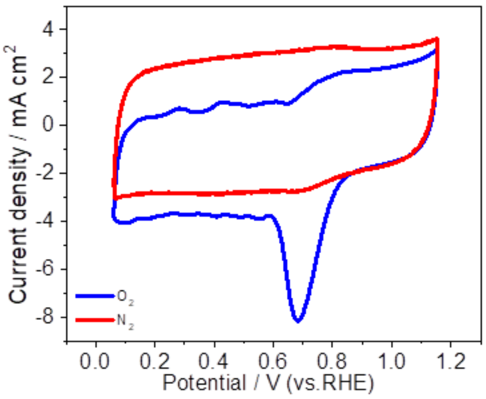

Fig. S22 Cyclic voltammograms of a GC electrode coated with the Fe/Co-CTs/NC catalyst (loading: 800 µg cm^{-2}), recorded in N_2- and O_2-saturated 0.1 M $HClO_4$ at a scan rate of 50 mV s^{-1}

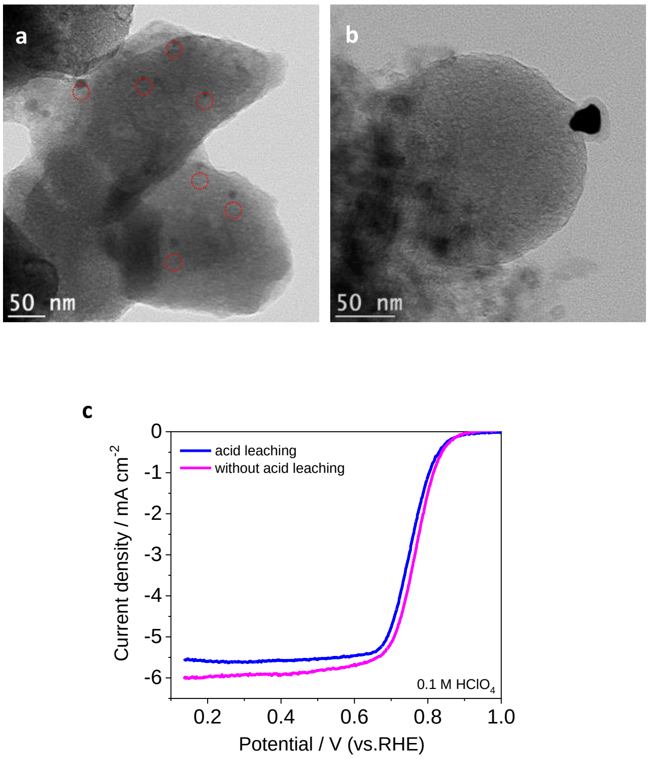

Fig. S23 a, b) TEM images of Fe/Co-CTs/NC with acid leaching. **c**) ORR polarization plots of Fe/Co-CTs/NC with and without acid leaching

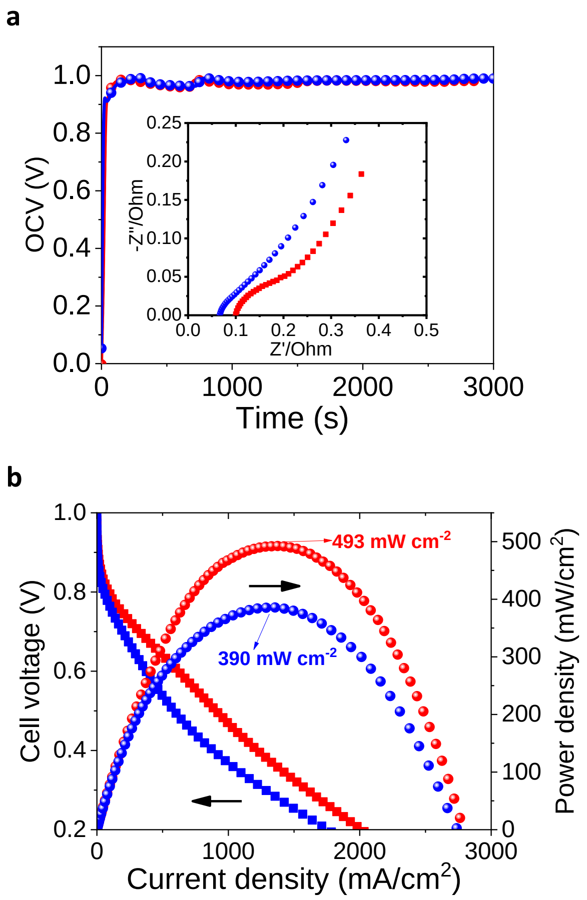

Fig. S24 a) Electrochemical impedance spectra (EIS) at an open-circuit voltage (OCV) of H_2/O_2 fuel cell measured at 80 °C using the as-prepared Fe/Co-CTs/NC (red) and Fe/Co-CTs/CNTs (blue). **b**) The electrochemical performance (I–V curves) of the H_2/O_2 fuel cell measured at 80 °C

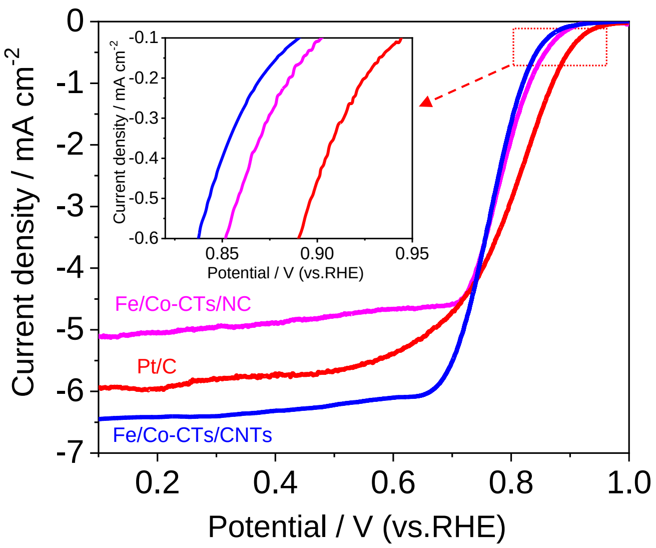

Fig. S25 ORR polarization plots of as-prepared samples (rotation rate: 1600 rpm) in O_2-saturated 0.1 M $HClO_4$ at a scan rate of 5 mV s^{-1}

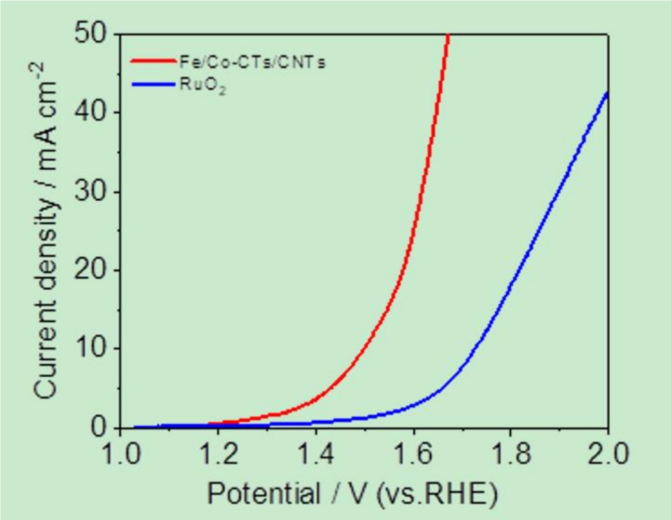

Fig. S26 Comparative OER activities of Fe/Co-CTs/CNTs and RuO_2 in 1.0 M KOH solution at 50 mV s^{-1}

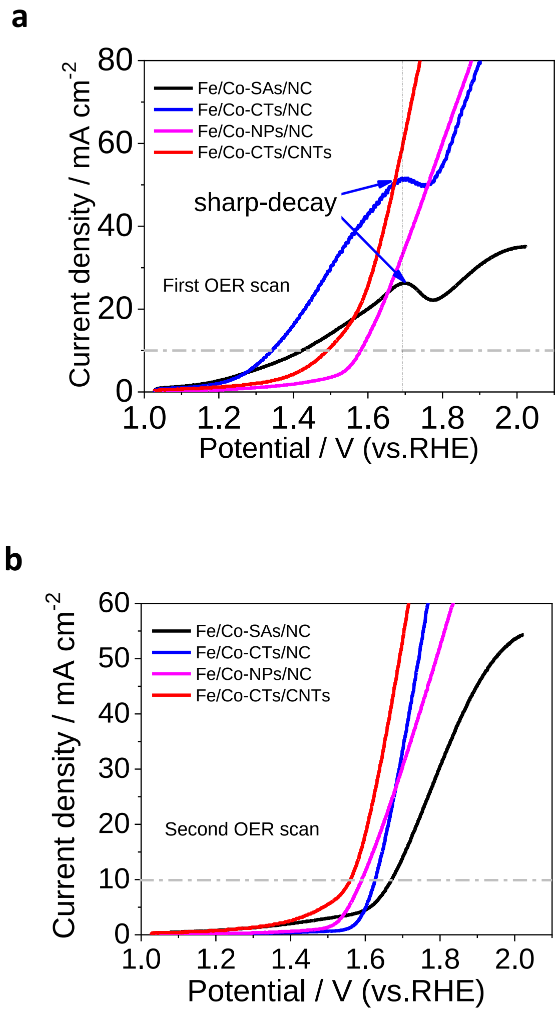

Fig. S27 a) OER polarization plots (First OER scan) of as-prepared samples. **b**) OER polarization plots (Second OER scan) of as-prepared samples

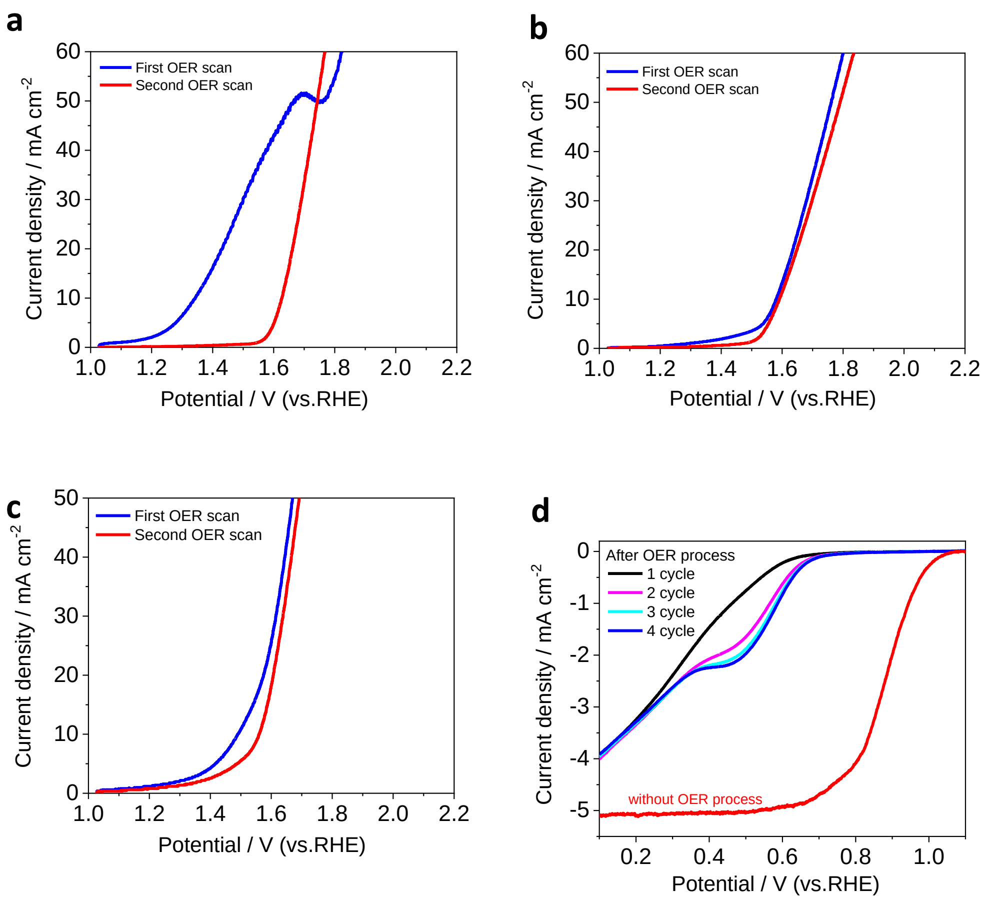

Fig. S28 OER polarization plots of **a**) Fe/Co-CTs/NC, **b**) Fe/Co-NPs/NC, and **c**) Fe/Co-CTs/CNTs in 1.0 M KOH. **d**) ORR polarization plots of Fe/Co-CTs/NC in 0.1 M KOH at a scan rate of 5.0 mV s^{-1}

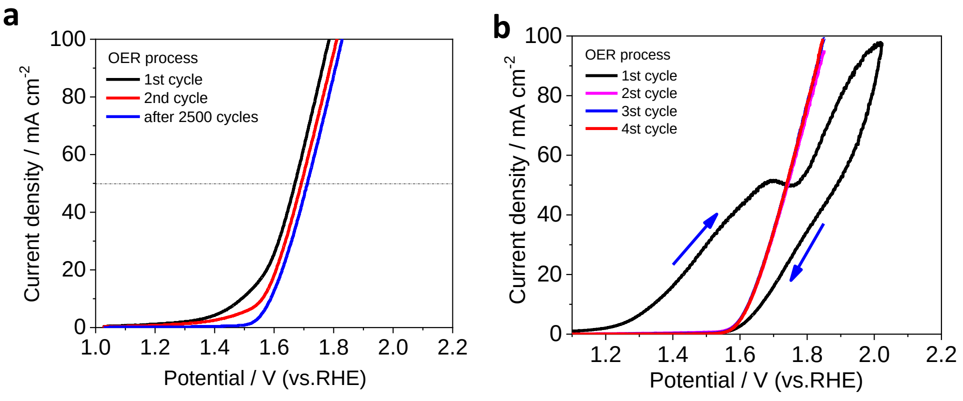

Fig. S29 a) Comparative OER activities of Fe/Co-CTs/CNTs before and after 2500 cycles of CV between 1.4 and 1.8 V in 1.0 M KOH solution at 50 mV s^{-1}. b) OER polarization plots of Fe/Co-CTs/NC in 1.0 M at a scan rate of 5.0 mV s^{-1}.

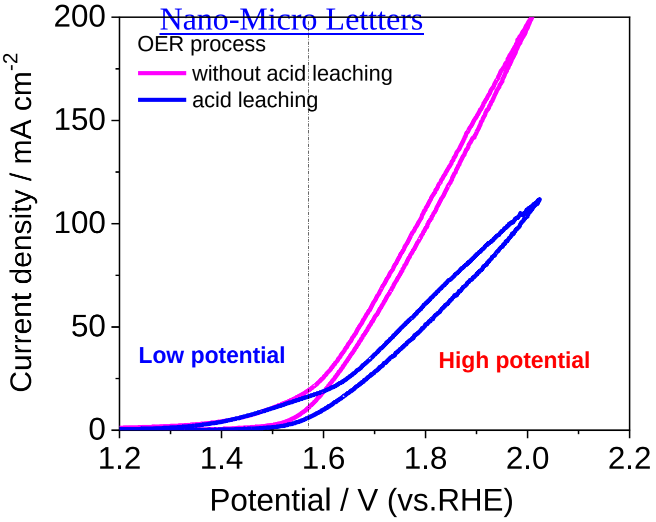

Fig. S30 a) OER polarization plots of Fe/Co-CTs/CNTs with and without acid leaching. **b**) Polarization curves of Fe/Co-CTs/CNTs before and after 1,000 cycles (-0.1 to -0.25 V. **c**) HER and OER polarization plots of Fe/Co-CTs/CNTs. (rotation rate: 1600 rpm, 1.0 M KOH, and a scan rate of 5.0 mV s^{-1}

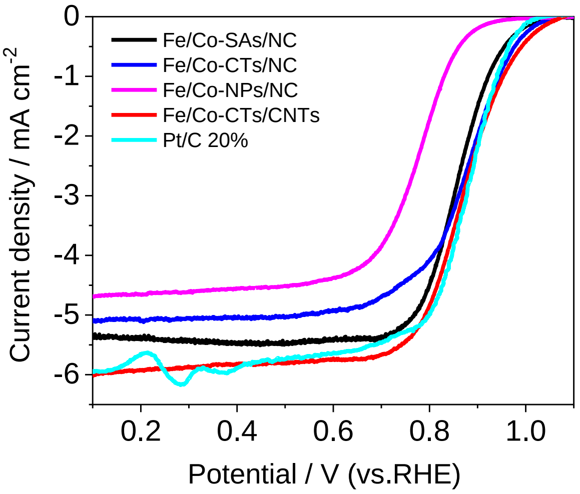

Fig. S31 ORR polarization plots of as-prepared samples (rotation rate: 1600 rpm) in O_2-saturated 0.1 M KOH at a scan rate of 5.0 mV s^{-1}

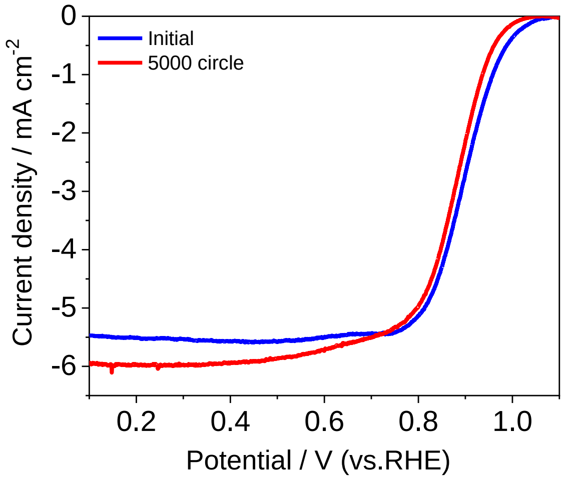

Fig. S32 Comparative OER activities of Fe/Co-CTs/CNTs before and after 5000 cycles of CV between 0.6 and 1.1 V in O_2-saturated 0.1 m KOH solution at 50 mV s^{-1}

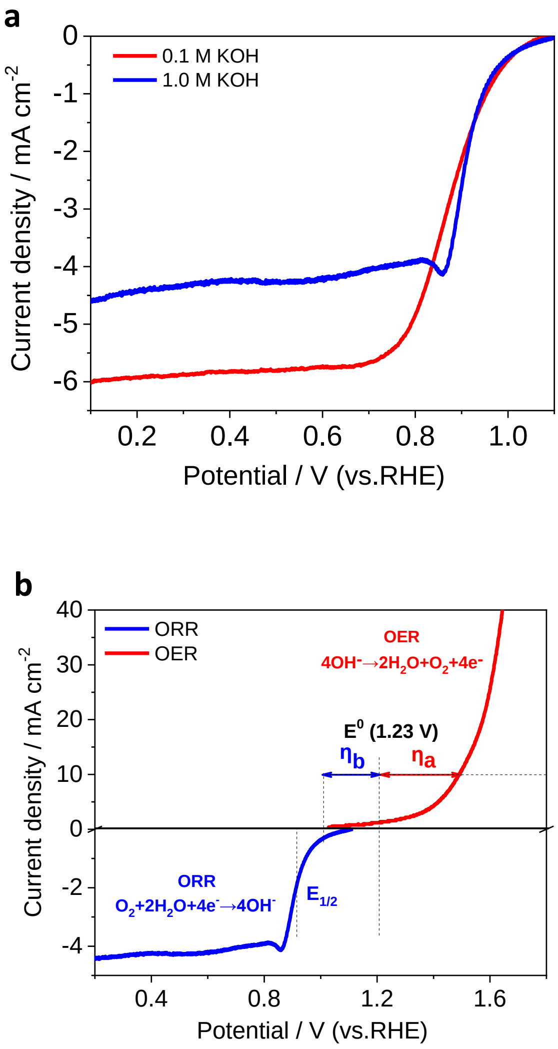

Fig. S33 a) ORR polarization plots of Fe/Co-CTs/CNTs (rotation rate: 1600 rpm) in O_2-saturated 0.1 M and 1.0 M KOH, respectively. **b**) ORR and OER polarization plots of Fe/Co-CTs/CNTs in 1.0 M KOH at a scan rate of 5 mV s^{-1}

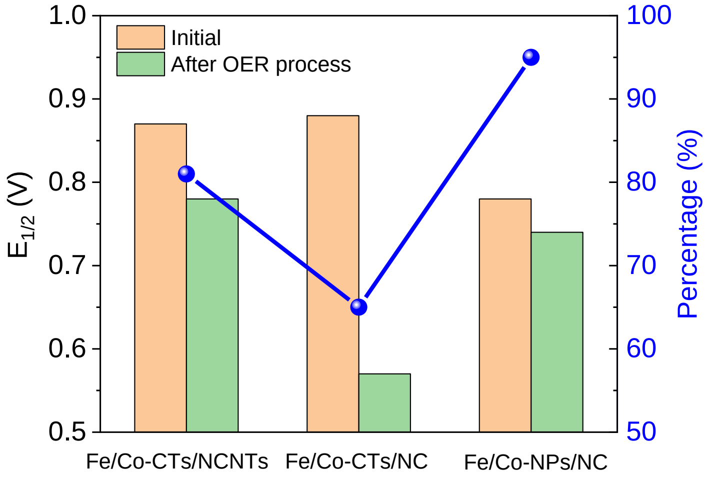

Fig. S34 Half-wave potential ($E_{1/2}$) before and after the OER process and the final activity retained percentage

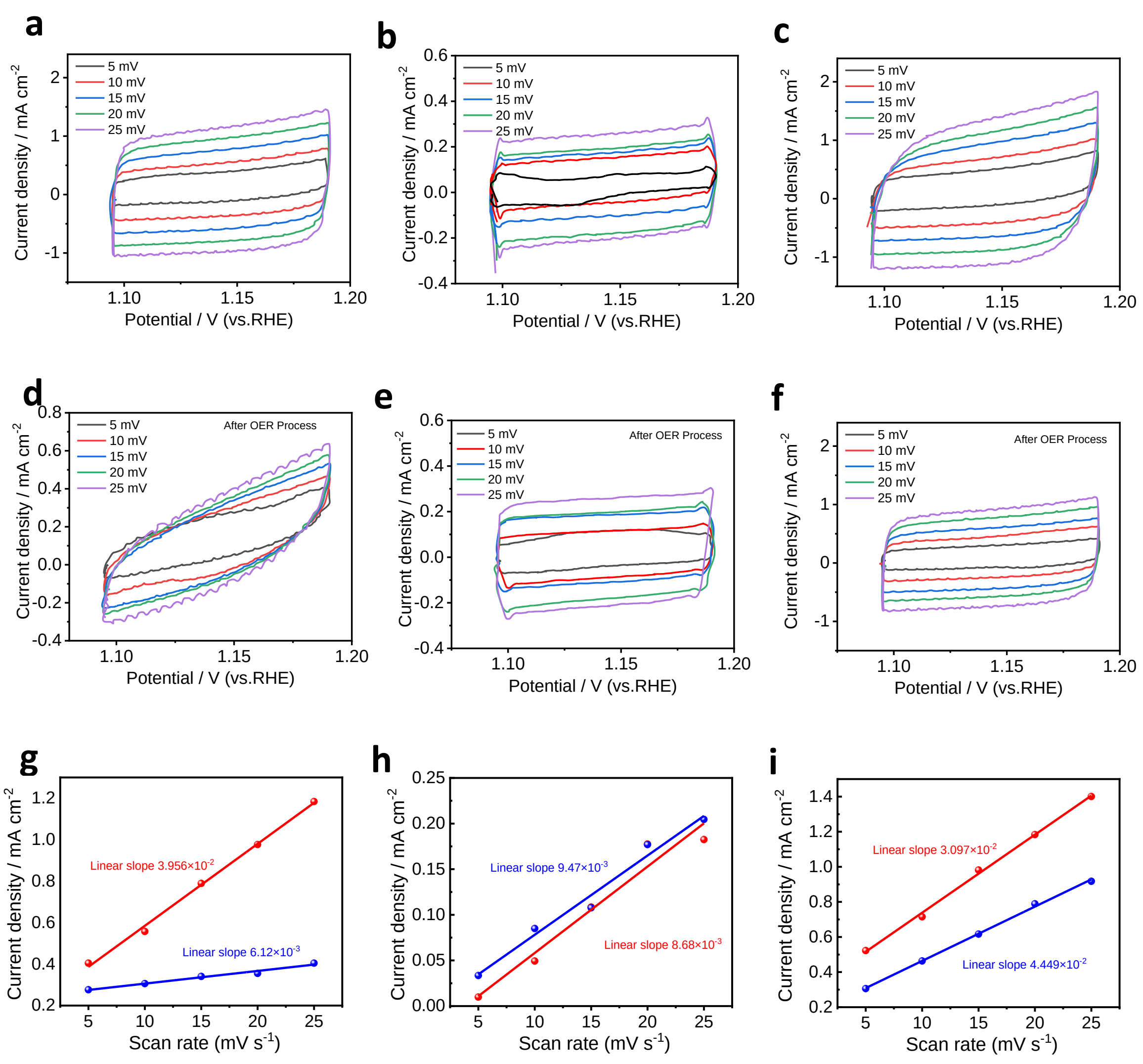

Fig. S35 CV curves of a) Fe/Co-CTs /NC, b) Fe/Co-NPs/NC and c) Fe/Co-CTs/CNTs at different scan rates. CV curves of d) Fe/Co-SAs/NC, e) Fe/Co-NPs/NC and f) Fe/Co-CTs/CNTs at different scan rates after the OER process. The capacitive current of g) Fe/Co-CTs/NC, h) Fe/Co-NPs/NC and i) Fe/Co-CTs/CNTs measured at 1.15 V versus RHE as a function of scan rate

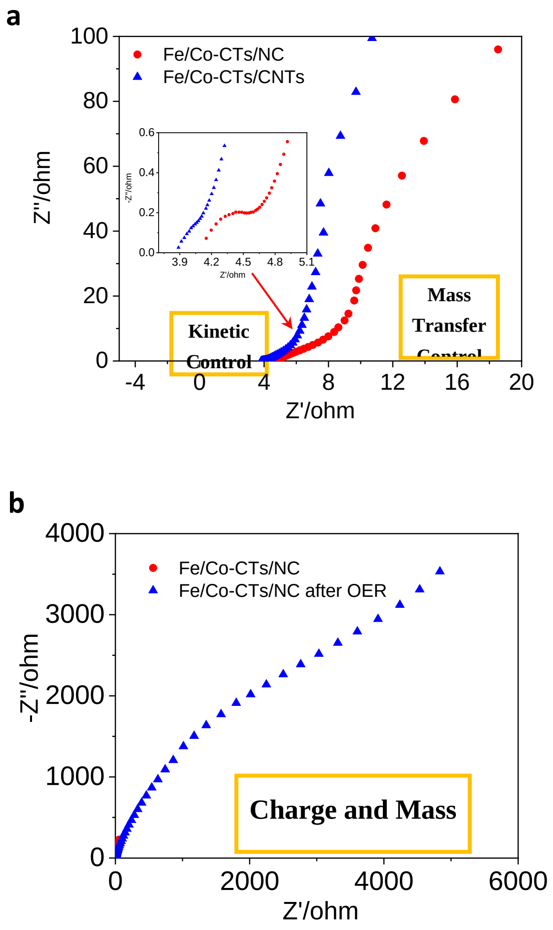

Fig. S36 a, b) Electrochemical impedance spectra (EIS) of various catalysts at a three-electrode system

Table S3 DFT model vs. R space curve fitting result of Fe/Co-SAs/NC before and after OER

	DFT model ($Fe-N_4$)				R space curve fitting		
	Path	CN	R		CN	R	DW
Before OE	Fe-N	4	1.87		4.0	1.95	0.0034
	Fe-C	4	2.65		4.0	2.53	0.0058
After OE	Fe-N	4	1.87		4.1	1.95	0.0011
	Fe-C	4	2.65		4.0	2.53	0.004

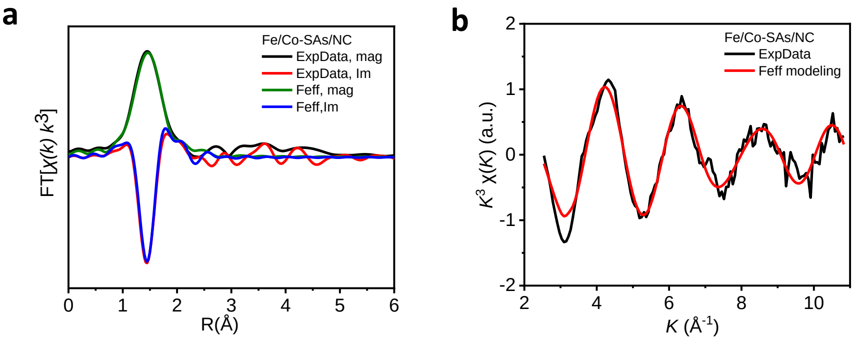

Fig. S37 Comparison is made for a) the magnitude and the imaginary part of Fourier transform between the experimental and the Feff modeling; and b) $k^3\chi(k)$ between the experimental and the Feff modeling based on the R space curve fitting result for the Fe/Co-SAs/NC after OER

The fitted parameters from the corresponding fitting are summarized in Table S3. OER enhanced the stability of the local structural environment FeN_4 configure, illustrated by significantly decreasing the DW parameters by 68% and 31% for paths Fe-N and Fe-C, respectively. But OER has no impact on the geometry of the FeN_4 configure and its nearest neighbor C coordination. c-d) Geometric structure of the FeN_4 sites before and after OER process.

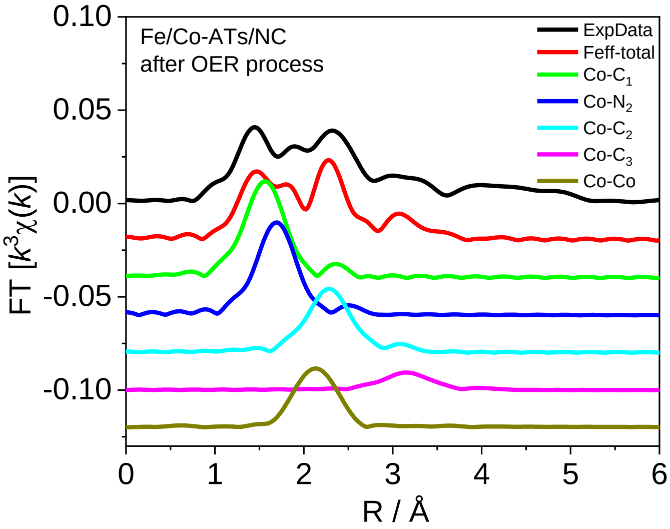

Fig. S38 Comparison is made for the magnitude and the imaginary part of Fourier transform between the experimental and the Feff modeling for the Fe/Co-SAs/NC after OER. The fitted parameters from the corresponding fitting are summarized in Table S4

Table S4 Structural models and R space fitting result to filter possible structures

Model	Structural model			Fit result			
	Path	CN	Description	CN	R	DW	E0/Residual
M-1	Co-N1	2	hybrid of simplified models Co-N4 and FeN4 (without O at this fitting stage), *ref.* *EXCEL-Mingjie-20190708;*	2.0	1.88	0.0043	-0.6
	Co-N2	2		2.2	2.13	0.0040	
	Co-Fe	1		1.2	2.53	0.0050	30.5
	Co-C	4 to 8	complex CoN4 and FeN4 are at identical nearest neighbored site; Bonds Co-O & Fe-O point to an opposite direction away from the graphene sheet	6.5	3.13	0.0050	
M-2	Co-N1	2	extension of M-1, i.e., N2 of CoN4 are replaced by C(×2) with constrain of Co-C≥Co-N	1.7	1.87	0.0038	0.3
	Co-C2	2		2.2	2.16	0.0012	
	Co-Fe	1		1.2	2.53	0.0050	39.2
	Co-C	4 to 8		7.1	3.13	0.0059	
M-3	Co-C1	2	extension of M-1, N1 of CoN4 are replaced by C(×2) with constrain of Co-C≤Co-N	2.2	1.92	0.0040	6
	Co-N2	2		2.2	2.19	0.0040	
	Co-Fe	1		1.2	2.54	0.0050	29
	Co-C	4 to 8		6.3	3.14	0.0050	
M-4	Co-N1	2	extension of M-1 with Fe replaced by Co, namely two CoN4 are closest neighbored	2	1.88	0.0045	-2
	Co-N2	2		1.9	2.13	0.0040	
	Co-Co	1		1.2	2.51	0.0050	31.1
	Co-C	4 to 8		9.8	3.15	0.0050	

	Path	CN		CN	R	DW	
M-5	Co-C1	2	hybrid of M3-4 & M-4	2.2	1.92	0.0040	5.2
	Co-N2	2		2	2.19	0.0040	
	Co-Co	1	N1(×2) of M-1 are replaced by C(×2) with constrain of Co-C≤Co-N	1.2	2.53	0.0050	
	Co-C	4 to 8	Fe of M-1 replaced by Co	10.3	3.17	0.0050	28

Table S5 Co R space curve fitting for Fe/Co-SAs/NC before and after OER

	M-8				R space curve fitting					
					before OER			after OER		
	No.	Path	CN	R	CN	R	DW	CN	R	DW
M-5	1	Co-C_1	2	1.84	1.8	1.82	0.0041	2.0	1.95	0.0015
	2	Co-N_2	2	1.98	1.8	1.91	0.0041	2.1	2.09	0.0015
	3	Co-C_2	4	2.65-2.66	4.2	2.82	0.0033	3.8	2.73	0.0033
	4	Co-C_3	CN≥4	3.01-3.02 for the 1st four C. For further outer C bonding, R is larger the specified value range	5.8	3.13	0.0033	2.2	3.63	0.0033
Metallic Co	5	Co-Co	1	2.50	1.2	2.55	0.0016	0.4	2.45	0.0023

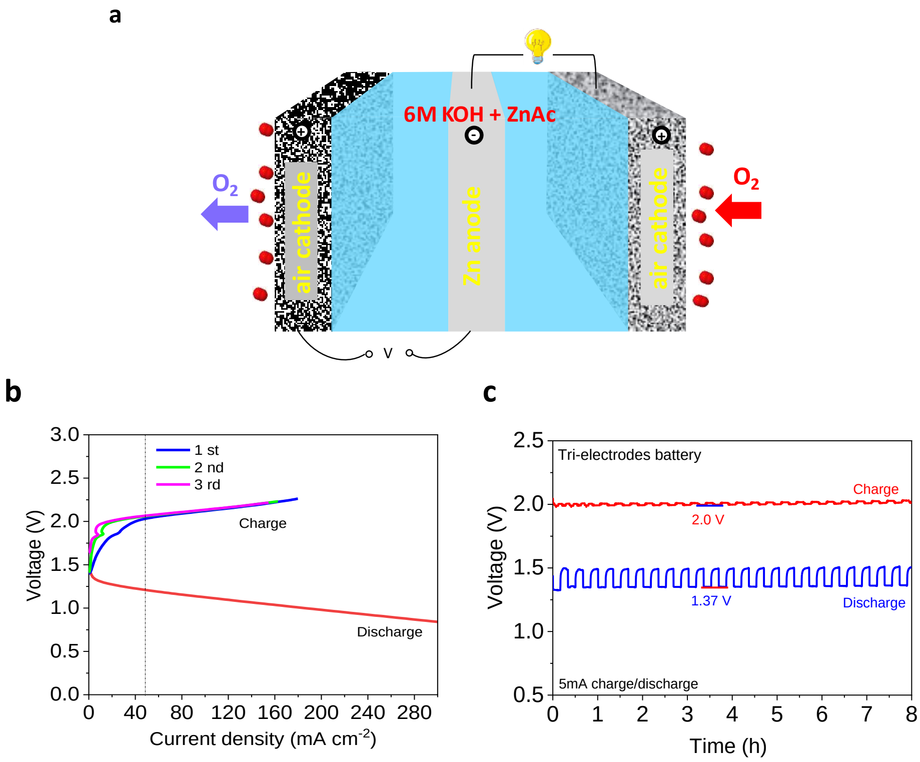

Fig. S39 a) A schematic of the tri-electrode configuration with Fe/Co-CTs/NC as ORR and OER catalysts for discharge and charge, respectively. **b**) Discharge and charge polarization

curves of tri-electrodes ZAB with Fe/Co-CTs/NC. **c**) Galvanostatic discharge and charge cycling stability of Fe/Co-CTs/NC at 5.0 mA cm^{-2}

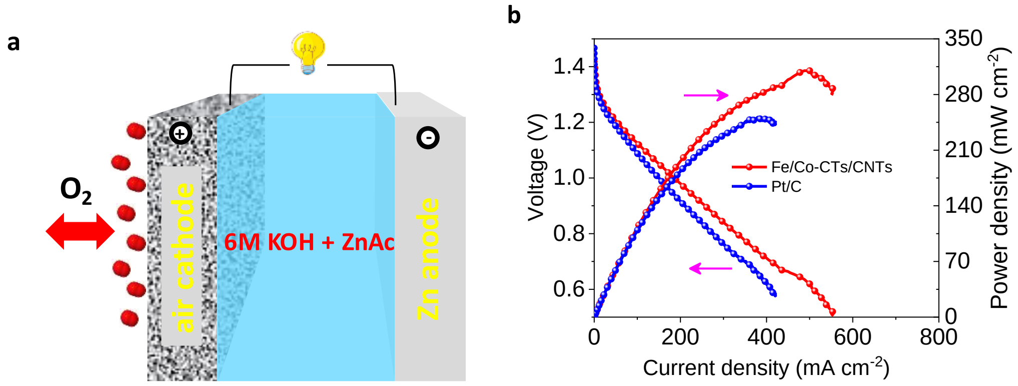

Fig. S40 a) A schematic structure of the two-electrode battery with Fe/Co-CTs/CNTs catalysts. **b**) A polarization curves (*V* ~ *i*) and corresponding power density plots of the liquid ZABs with Fe/Co-CTs/CNTs and Pt/C, respectively. The battery had an open circuit voltage of 1.41 V. The peak power density was 310 mW cm^{-2} outperformed the battery made with Pt/C

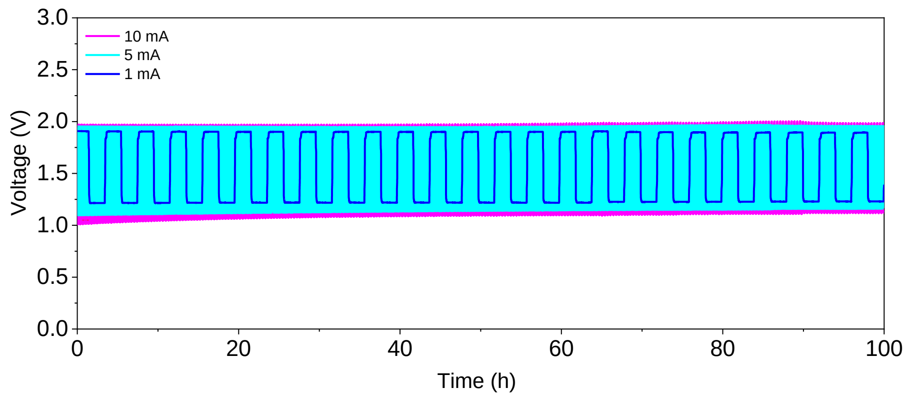

Fig. S41 Galvanostatic discharge and charge cycling stability of **a**) Fe/Co-CTs/NC and **b**) Fe/Co-CTGalvanostatic discharge and charge cycling stability of Fe/Co-CTs/CNTs at different current densities. Electrolyte: 6.0 M KOH with 0.2 M zinc acetate. The battery of Fe/Co-CTs/CNTs exhibited well-cycling stability at different current densities

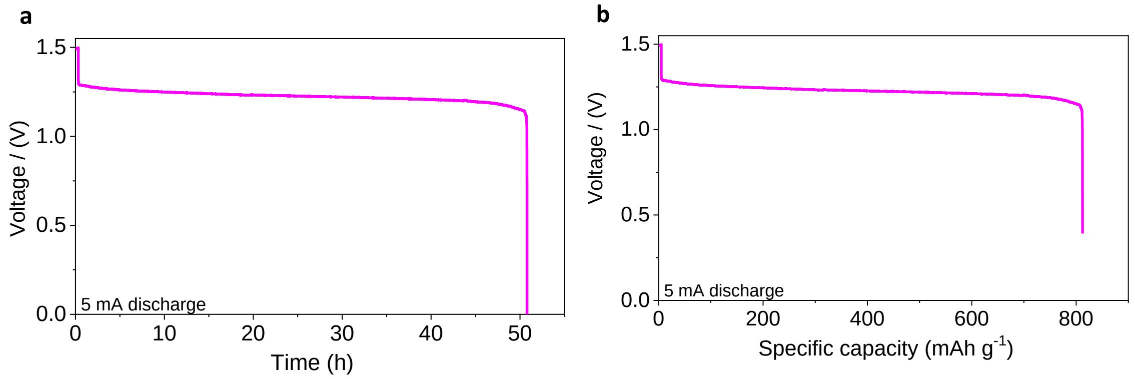

Fig. S42 a) Long-time galvanostatic discharge curves of Fe/Co-CTs/CNTs until complete consumption of Zn at a constant current density of 5.0 mA cm^{-2}. **b**) The corresponding specific capacity curves. The specific capacity at 5.0 mA cm^{-2} is 812 mAh g^{-1}, corresponding to a high energy density of 922 W h kg^{-1}

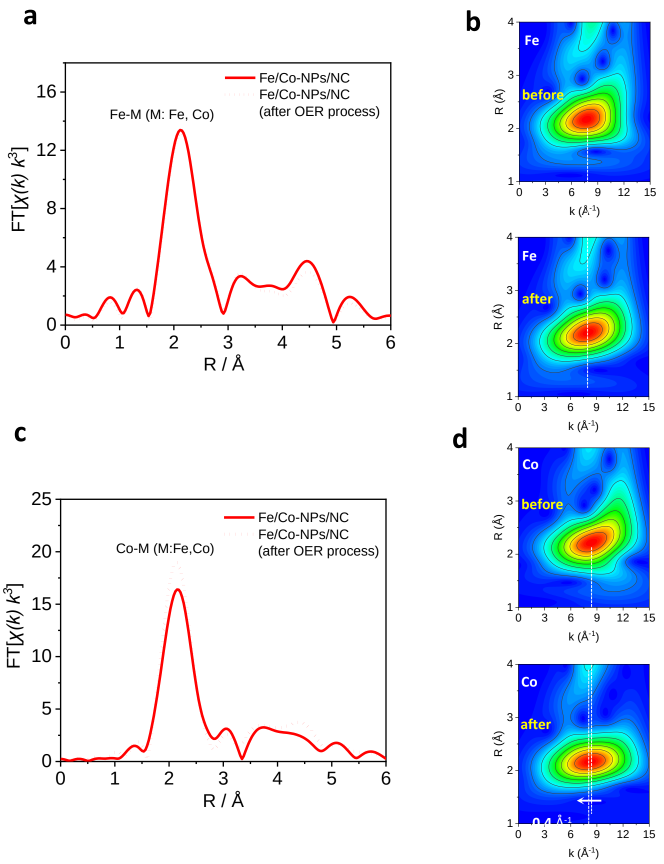

Fig. S43 The corresponding Fourier transforms of **a**) Fe and **c**) Co K-edge EXAFS oscillations $k^3\chi(k)$ of Fe/Co-NPs/NC (k-weight: 3). **b**) Fe and **d**) Co K-edge WT-EXAFS contour plots of Fe/Co-SAs/NC before and after OER process

Table S6 Comparison of the ORR activity of present work with the literature reporte bifunctional catalysts in acid

Catalysts	$E_{1/2}$ (V)	Reference
Fe_3C/NG-800	0.77 V(0.1 M $HClO_4$)	[S4]
Fe_3C/C-700	0.73 V(0.1 M $HClO_4$)	[S5]
Fe−N/C-800	0.62 V(0.1 M $HClO_4$)	[S6]
Fe_2N/N-GAs-20	0.71 V (1.0 M $HClO_4$)	[S7]
Fe-N-CNFs	0.61V (0.5 M H_2SO_4)	[S8]
Fe/N/CF	0.80 V (0.5 M H_2SO_4)	[S9]
PANI-Fe-C	0.80 V (0.5 M H_2SO_4)	[S10]
**Fe/Co-CTs/NC**	0.79 V(0.1 M $HClO_4$)	**This work**
**Fe/Co-CTs/CNTs**	0.77 V (0.1 M $HClO_4$)	

Table S7 Comparison of the ORR/OER activity of present work with the literature reported bifunctional catalysts in alkaline

Catalysts	$E_{j=10}$ (V)	$E_{1/2}$ (V)	ΔE (V)	E_{onset} (ORR)	Reference
Co-MOF	1.45, 1.0 M KOH	0.7, 1.0 M KOH	0.75	--	[S11]
Fe-N_4 SAs/NPC	1.66, 1.0 M KOH	0.88, 0.1 M KOH	0.78	0.97	[S12]
$Fe_{0.5}Ni_{0.5}$@N-GR	1.44, 1.0 M KOH	0.83, 0.1 M KOH	0.61	0.94	[S13]
Fe@C–NG/CNTs	1.68, 1.0 M KOH	0.84, 0.1 M KOH	0.84	0.93	[S14]
Ni, N-graphene	1.50, 1.0 M KOH	0.85, 0.1 M KOH	0.66	0.99	[S15]
Mn/Fe-HIB-MOF	1.51, 1.0 M KOH	0.88, 0.1 M KOH	0.63	0.98	[S16]
NC-Co_3O_4-90	1.58, 1.0 M KOH	0.87, 0.1 M KOH	0.71	0.91	[S17]
Co SA@NCF/CNF	1.63, 1.0 M KOH	0.88, 0.1 M KOH	0.75	--	[S18]
**Fe/Co-CTs/NC**	**1.35, 1.0 M KOH**	**0.88, 0.1 M KOH**	**0.47 (not stable)**	**1.03**	**This work**
**Fe/Co-CTs/CNTs**	**1.50, 1.0 M KOH**	**0.87, 0.1 M KOH**	**0.63**	**1.05**	
	**1.50, 1.0 M KOH**	**0.90, 1.0 M KOH**	**0.60**	**1.06**	

Supplementary References

[S1] M. Wu, Q. Wei, G. Zhang, J. Qiao, M. Wu et al. Fe/Co Double Hydroxide/Oxide Nanoparticles on N-Doped CNTs as Highly Efficient Electrocatalyst for Rechargeable Liquid and Quasi-Solid-State Zinc-Air Batteries. Advanced Energy Materials, 1801836 (2018). https://doi:10.1002/aenm.201801836

[S2] A. Lu, Y. Li, H. Ding, X. Xu, Y. Li et al. Photoelectric conversion on Earth's surface via widespread Fe- and Mn-mineral coatings. Proc Natl Acad Sci USA **116**, 9741-9746 (2019). https://doi:10.1073/pnas.1902473116

[S3] M.J. Wu, G.X. Zhang, J.L. Qiao, N. Chen, W.F. Chen et al. Ultra-long life rechargeable zinc-air battery based on high-performance trimetallic nitride and NCNT hybrid bifunctional electrocatalysts. Nano Energy **61**, 86-95 (2019). https://doi:10.1016/j.nanoen.2019.04.031

[S4] M. Xiao, J. Zhu, L. Feng, C. Liu & W. Xing. Meso/Macroporous Nitrogen-Doped Carbon Architectures with Iron Carbide Encapsulated in Graphitic Layers as an Efficient and Robust Catalyst for the Oxygen Reduction Reaction in Both Acidic and Alkaline Solutions. Adv. Mater. **27**, 2521-2527 (2015). https://doi:10.1002/adma.201500262

[S5] Y. Hu, J.O. Jensen, W. Zhang, L.N. Cleemann, W. Xing et al. Hollow spheres of iron carbide nanoparticles encased in graphitic layers as oxygen reduction catalysts. Angew. Chem. Int. Ed. Engl. **53**, 3675-3679 (2014). https://doi:10.1002/anie.201400358

[S6] 6 L. Lin, Q. Zhu & A.W. Xu. Noble-metal-free Fe-N/C catalyst for highly efficient oxygen reduction reaction under both alkaline and acidic conditions. J. Am. Chem. Soc. **136**, 11027-11033 (2014). https://doi:10.1021/ja504696r

[S7] L. Liu, X. Yang, N. Ma, H. Liu, Y. Xia et al. Scalable and Cost-Effective Synthesis of Highly Efficient Fe_2N-Based Oxygen Reduction Catalyst Derived from Seaweed Biomass. Small **12**, 1295-1301 (2016). https://doi:10.1002/smll.201503305

[S8] Z.Y. Wu, X.X. Xu, B.C. Hu, H.W. Liang, Y. Lin et al. Iron Carbide Nanoparticles Encapsulated in Mesoporous Fe-N-Doped Carbon Nanofibers for Efficient Electrocatalysis. Angew. Chem. Int. Ed. Engl. **54**, 8179-8183 (2015). https://doi:10.1002/anie.201502173

[S9] J. Shui, C. Chen, L. Grabstanowicz, D. Zhao & D.J. Liu. Highly efficient nonprecious metal catalyst prepared with metal-organic framework in a continuous carbon nanofibrous network. Proc Natl Acad Sci USA **112**, 10629-10634 (2015). https://doi:10.1073/pnas.1507159112

[S10] G. Wu, K.L. More, C.M. Johnston & P. Zelenay. High-performance electrocatalysts for oxygen reduction derived from polyaniline, iron, and cobalt. Science **332**, 443-447 (2011). https://doi:10.1126/science.1200832

[S11] G. Chen, J. Zhang, F. Wang, L. Wang, Z. Liao et al. Cobalt-Based Metal-Organic Framework Nanoarrays as Bifunctional Oxygen Electrocatalysts for Rechargeable Zn-Air Batteries. Chemistry **24**, 18413-18418 (2018). https://doi:10.1002/chem.201804339

[S12] Y. Pan, S. Liu, K. Sun, X. Chen, B. Wang et al. A Bimetallic Zn/Fe Polyphthalocyanine-Derived Single-Atom Fe-N_4 Catalytic Site:A Superior Trifunctional Catalyst for Overall Water Splitting and Zn-Air Batteries. Angew. Chem. Int. Ed. Engl. **57**, 8614-8618 (2018). https://doi:10.1002/anie.201804349

[S13] P. Liu, D. Gao, W. Xiao, L. Ma, K. Sun et al. Self-Powered Water-Splitting Devices by Core-Shell NiFe@N-Graphite-Based Zn-Air Batteries. Adv. Funct. Mater. **28**, 1706928 (2018). https://doi:10.1002/adfm.201706928

[S14] Q. Wang, Y. Lei, Z. Chen, N. Wu, Y. Wang et al. Fe/Fe_3C@C nanoparticles encapsulated in N-doped graphene–CNTs framework as an efficient bifunctional oxygen electrocatalyst for robust rechargeable Zn–air batteries.

Journal of Materials Chemistry A **6**, 516-526 (2018). https://doi:10.1039/c7ta08423d

[S15] H.J. Qiu, P. Du, K. Hu, J. Gao, H. Li et al. Metal and Nonmetal Codoped 3D Nanoporous Graphene for Efficient Bifunctional Electrocatalysis and Rechargeable Zn-Air Batteries. Adv. Mater., e1900843 (2019). https://doi:10.1002/adma.201900843

[S16] S.S. Shinde, C.H. Lee, J.-Y. Jung, N.K. Wagh, S.-H. Kim et al. Unveiling dual-linkage 3D hexaiminobenzene metal–organic frameworks towards long-lasting advanced reversible Zn-air batteries. Energy & Environmental Science **12**, 727-738 (2019). https://doi:10.1039/c8ee02679c

[S17] C. Guan, A. Sumboja, H. Wu, W. Ren, X. Liu et al. Hollow Co_3O_4 Nanosphere Embedded in Carbon Arrays for Stable and Flexible Solid-State Zinc-Air Batteries. Adv. Mater. **29** (2017). https://doi:10.1002/adma.201704117

[S18] D. Ji, L. Fan, L. Li, S. Peng, D. Yu et al. Atomically Transition Metals on Self-Supported Porous Carbon Flake Arrays as Binder-Free Air Cathode for Wearable Zinc-Air Batteries. Adv. Mater. **31**, e1808267 (2019). https://doi:10.1002/adma.201808267
